# Immobilization stress-induced *Escherichia coli* causes anxiety by inducing NF-κB activation through gut microbiota disturbance

**DOI:** 10.1038/s41598-018-31764-0

**Published:** 2018-09-17

**Authors:** Hyo-Min Jang, Kyung-Eon Lee, Hae-Ji Lee, Dong-Hyun Kim

**Affiliations:** Neurobiota Research Center and Department of Life and Nanopharmaceutical Sciences, College of Pharmacy, Kyung Hee University, 26, Kyungheedae-ro Dongdaemun-gu, Seoul, 02447 Korea

## Abstract

The present study aimed to understand the crosstalk between anxiety and gut microbiota. Exposure of mice to immobilization stress (IS) led to anxiety-like behaviors, increased corticosterone and tumor necrosis factor-α levels in the blood, increased nuclear factor (NF)-κB activation and microglia/monocyte populations in the hippocampus, and suppressed brain-derived neurotrophic factor (BDNF) expression in the hippocampus. Furthermore, IS exposure increased NF-κB activation and monocyte population in the colon and increased Proteobacteria and *Escherichia coli* populations in the gut microbiota and fecal and blood lipopolysaccharide (LPS) levels while decreasing the *lactobacilli* population. Oral administration of the fecal microbiota of mice treated with IS (FIS) or *E*. *coli* led to the increased NF-κB activation and monocyte population in the colon. These treatments increased blood corticosterone and LPS levels and anxiety-like behaviors, decreased BDNF expression, and induced NF-κB activation and microglia/monocyte populations in the hippocampus. Intraperitoneal injection of LPS purified from *E*. *coli* also led to anxiety and colitis in mice. Oral administration of commensal *lactobacilli*, particularly *Lactobacillus johnsonii*, attenuated IS- or *E*. *coli*-induced colitis and anxiety-like behaviors and biomarkers. These findings suggest that exposure to stressors can increase Proteobacteria populations and fecal LPS levels and cause gastrointestinal inflammation, resulting in the deterioration of anxiety through NF-κB activation. However, the amelioration of gastrointestinal inflammation by treatment with probiotics including *L*. *johnsonii* can alleviate anxiety.

## Introduction

Anxiety disorders are the most prevalent mental disorders, affecting up to 10% of the world’s population^1–3^. Anxiety was raised by the exposure to stressors such as immobilization, forced swimming, social defeat, and inescapable tail-shock^4^. The exposure to stressors as such led to the secretion of adrenal hormones such as adrenaline, noradrenaline, and glucocorticoids via the hypothalamo-pituitary-adrenal (HPA) axis and the modulation of the cytokine expression in immune cells^5–8^; and disturbance of the gut microbiota composition^9–11^. Gut microbiota, which consist of bacteria, viruses, protozoa, archaea, and fungi in the mammalian intestinal tract, stimulate the immune and central nervous systems (CNS) via the gastrointestinal (GI) tract^12,13^; this interaction forms a microbiota-gut-brain (MGB) axis^14,15^. Gut microbes play a role in host physiology through their contribution to nutrient and xenobiotic metabolites (e.g., vitamins, polysaccharide metabolites, and drug metabolites), microbial byproducts (e.g., short chain fatty acids and lipopolysaccharide [LPS]), immune cytokines (e.g., interleukin [IL]-6 and tumor necrosis factor [TNF]-α), neuroendocrine hormones (cortisol), and neurotransmitters (e.g., norepinephrine, dopamine, and gamma-aminobutyric acid)^16–19^. These influence the gut barrier, inflammatory response, and metabolic homeostatic control in different tissues^18,20^. Therefore, gut microbiota disturbance (dysbiosis) is associated with not only GI diseases such as inflammatory bowel disease but also systemic diseases such as obesity, autoimmune arthritis, and psychiatric disorders, including schizophrenia, autism, anxiety, and depression^19–21^. For instance, dysbiosis accelerates the occurrence of anxiety and depressive disorders by regulating the expression, secretion, and turnover of neurotransmitters in nervous systems^22–24^ and cytokines in the GI tract^25,26^. Microbial establishment in the GI tract is important in the development and maturation of both the enteric nervous system (ENS) and CNS^22,27^. Germ-free mice displayed more hyperactive anxiety-like behaviors than did specific pathogen-free (SPF) mice^24^. However, the absence of the gut microbiota (germ-free) enhanced anxiety-like behaviors and neuroendocrine response to acute stress^28^. Furthermore, transplantation of gut microbiota or *Bifidobacteria* into germ-free mice suppressed anxiety-like behaviors and up-regulated brain-derived neurotrophic factor (BDNF) expression in the hippocampus^24,29^. In addition, exposure to stressors in experimental animals increases adrenal hormone levels and caused gut microbiota disturbance, resulting in anxiety^30–33^. Thus, the disruption of MGB axis is associated with the increased occurrence of psychiatric disorders such as anxiety and depression. Nevertheless, studies on the role of gut bacteria in the psychiatric disorders remain elusive.

In the present study, to understand whether stress-induced gut microbiota could raise anxiety and what kinds of gut bacteria could alleviate or deteriorate anxiety, we investigated the gut microbiota composition in immobilization stress (IS)-treated mice, isolated commensal bacteria in the feces, and assessed their anxiety-like or anxiolytic effects in mice.

## Results

### Exposure of mice to IS raised anxiety and colitis and disturbed gut microbiota composition

**Figure 1:**
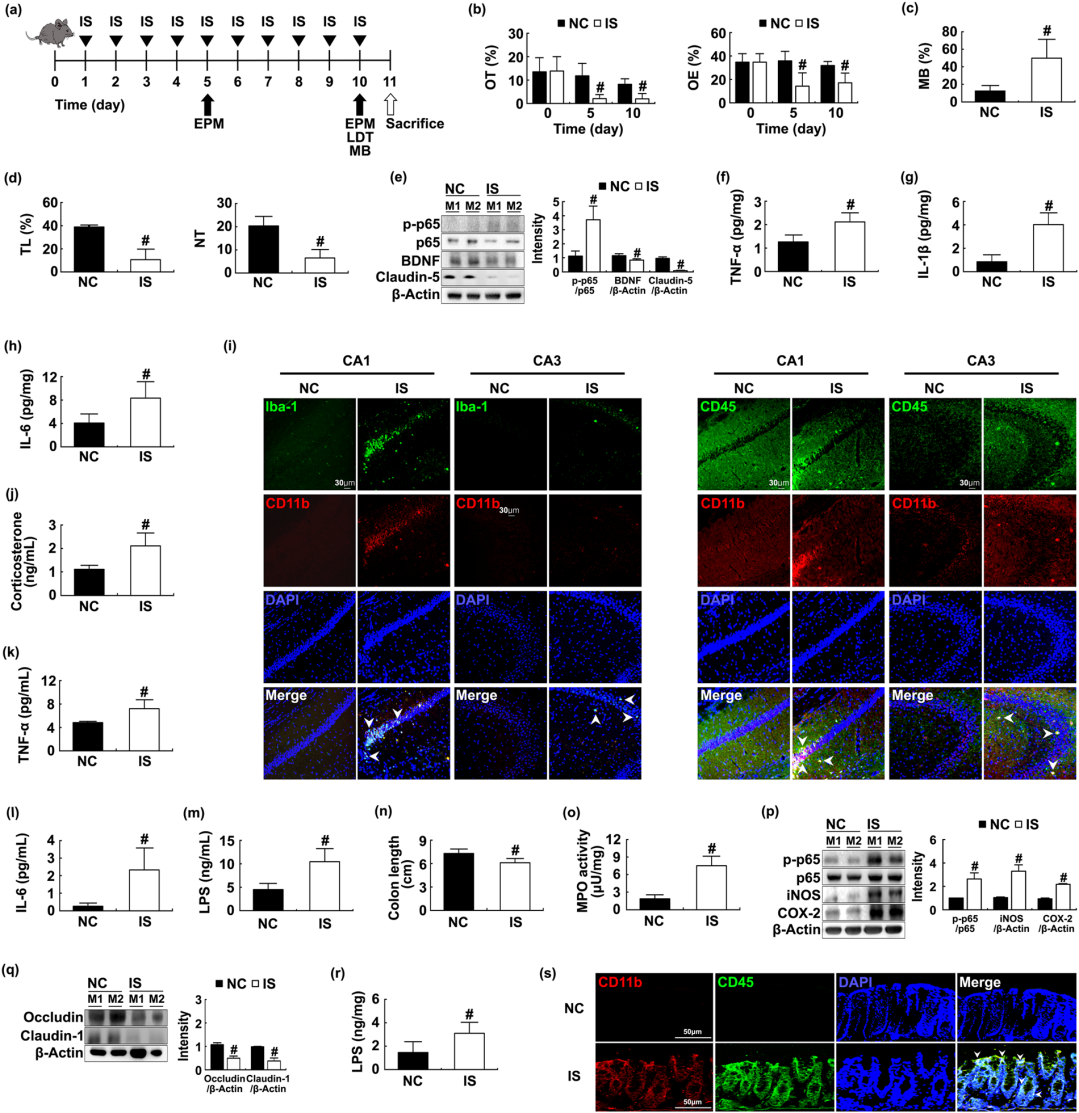
Immobilization stress (IS) induced anxiety-like behaviors and colitis in mice. Experimental time (**a**). Anxiety-like behaviors of mice were measured in the EPM (**b**) (time spent in open arms [OT] and open arm entries [OE]), MB (marble buried, **c**) and LDT (**d**, time spent in light area [TL] and number of transitions [NT]). Control mice (NC) were not treated with IS. NF-κB (p-p65 and p65), BDNF, claudin-5, and β-actin were measured in the hippocampus by immunoblotting (**e**). Brain TNF-α (**f**) IL-1β (**g**) and IL-6 levels (**h**) were analyzed by ELISA kit. Iba-1^+^/CD11b^+^ and CD11b^+^/CD11b^+^ populations were assayed in CA1 and CA3 regions of the hippocampus by a confocal microscope (**i**). Blood corticosterone (**j**) TNF-α (k), IL-6 (**l**) and LPS levels (**m**) were analyzed by ELISA or LAL kit. Colitis markers colon length (**n**) and myeloperoxidase (MPO) activity (**o**) were measured in the colon. Colonic iNOS and COX-2 expression, NF-κB activation (**p**) and tight junction proteins (occludin and claudin-1), and β-actin (**q**) were analyzed by immunoblotting. Fecal LPS level (**r**) and CD11b^+^/CD11b^+^ population (**s**) were analyzed by ELISA kit and confocal microscope (**i**) respectively. All data except immunoblotting data (n = 4) were expressed as mean ± SD (n = 8). ^#^*p* < 0.05 vs. NC group.

First, in order to understand the interaction between anxiety disorders and gut microbiota disturbance, we instituted IS once daily for 10 days and assessed anxiety-like behaviors in the elevated plus maze (EPM), marble-burying (MB), and light/dark transition (LDT) tasks (Fig. 1a–d). Exposure of mice to IS for 10 days led to gradually less time spent in the open arms (OT) and open arm entries (OE) in EPM task; it significantly decreased OT and OE by 74.5% [F(1,14) = 30.193, *p* < 0.001] and 47.4% [F(1,14) = 22.877, *p* < 0.001], respectively, of that spent by mice not treated with IS. IS exposure also increased anxiety-like behaviors in MB and LDT tasks. IS exposure suppressed BDNF and a tight junction protein claudin-5 expression and increased NF-κB activation (p-p65) and IL-6, TNF-α, and IL-1β expression in the hippocampus compared to control group not treated with IS (Fig. 1e–h). Furthermore, exposure to IS induced the infiltration of activated microglia (Iba^+^) and monocytes (CD11b^+^/CD45^+^) into the hippocampus, particularly the cornu ammonis 3 (CA3) region (Figs 1i and S1a). However, few of Iba+ and CD11b/CD45 populations in the cortex of brain were observed. Exposure to IS also led to corticosterone, IL-6, TNF-α, IL-1β, and LPS levels in the blood being 1.9 [F(1,14) = 32.620, *p* < 0.001], 6.7 [F(1,14) = 28.467, *p* < 0.001], 1.5 [F(1,14) = 26.117, *p* < 0.001], 2.3 F(1,14) = 355.129, *p* < 0.001], and 2.3 [F(1,14) = 46.084, *p* < 0.001] times higher, respectively, than those in control group (Figs 1j–m and S1b). Next, we measured colitis-associated biomarkers in IS-treated mice. Exposure to IS caused colitis; it induced colon shortening [F(1,14) = 19.243, *p* = 0.001] and myeloperoxidase activity [F(1,14) = 301.258, *p* < 0.001] and up-regulated nuclear factor (NF)-κB activation (p-p65) and cyclooxygenase (COX)-2, inducible nitric oxide synthase (iNOS), IL-6, TNF-α, and IL-1β expression in the colon (Figs 1n–q and S1c–h). Exposure to IS suppressed the expression of IL-10, occludin, and claudin-1 in the colon. Exposure to IS induced the infiltration of CD11b^+^/CD45^+^ populations, which might be composed of macrophages, dendritic cells, and neutrophils, into the colon, and increased the fecal LPS levels [F(1,14) = 25.920, *p* < 0.001] (Fig. 1r,s).

### IS increased the Proteobacteria population in mice

To investigate whether IS exposure disturbed the gut microbiota composition, we exposed IS to mice and measured the fecal bacterial populations by using qPCR. Exposure to IS significantly increased the Proteobacteria population [F(1,14) = 7.384, *p* = 0.017] while the Actinobacteria and Bacteroidetes populations were decreased (Supplement Fig. S1i).

**Figure 2:**
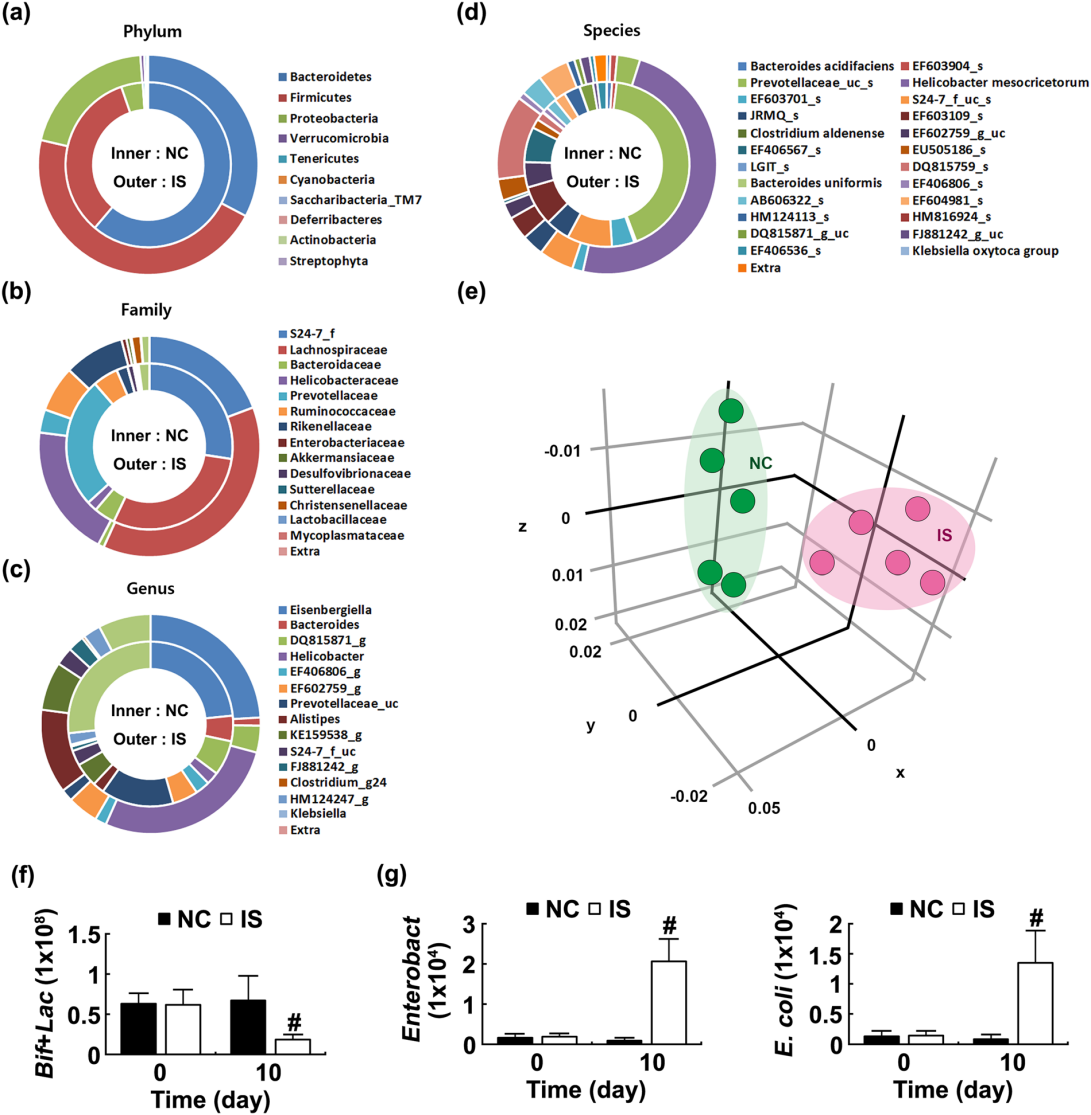
Effects of immobilization stress (IS) on the fecal microbiota composition in mice. The composition of gut microbiota was analyzed using the pyrosequencing: (**a**) phylum, (**b**) family, (**c**) genus, and (**d**) species. (**e**) Gut microbiota composition was analyzed by using principal coordinate analysis (PCoA) plot. The plot shows the clustering pattern between mice treated with IS (IS) and not treat with IS (NC) based on weighted pairwise Fast UniFrac analysis. All data were expressed as mean ± S.D. (n = 5). Gut microbiota composition of mice were also analyzed by the selective media (**f**) BL agar plates selective for *Bifidobacteria* [Bif] and *Lactobacilli* [Lac] and (**g**) DHL agar plates selective for *Enterobacteriaceae* [Enterobact]). All data were expressed as mean ± S.D. (n = 8). All data were expressed as mean ± SD (n = 8). ^#^*p* < 0.05 vs. NC group.

In order to verify the disintegration of gut microbiota composition by IS exposure, we assessed the composition of mouse fecal microbiota by using pyrosequencing (Fig. 2). The number of sequences analyzed, estimated operational taxonomic unit (OTU) richness, and coverage indicated that the richness and diversity of bacteria in IS-treated mice were significantly lower than in control mice. Exposure to IS significantly increased the Proteobacteria population at the phylum level while decreasing the Bacteroidetes population (Fig. 2a–d). At the family level, IS treatment significantly increased *Helicobacteraceae and Enterobacteriaceae* populations while *Lactobacillaceae* population was reduced. At the genus level, IS treatment increased *Klebsiella* sp and *Helicobacter* sp populations. Thereafter, these sequences were processed to match the length and position of mouse fecal microbiota 16S rRNA gene sequences analyzed by pyrosequencing, all pair-wise distances between mice treated with IS and not treated with IS were computed, and principal coordinate analysis was conducted, as previously reported. The community of control mouse gut microbiota differed significantly from IS-treated mouse ones (Fig. 2e).

Next, to search gut bacteria associated with the occurrence of anxiety in mice, we analyzed the fecal microbiota of mice treated or not treated with IS by culturing in the selective media (Fig. 2f,g). IS exposure increased the number of *Enterobacteriaceae* [F(1,14) = 99.046, *p* < 0.001] cultured in DHL agar plates, including *Escherichia coli* [F(1,14) = 44.038, *p* < 0.001], while the number of *Bifidobacteria* and *Lactobacilli* [F(1,14) = 19.347, *p* = 0.001] cultured in BL agar plates, including *Lactobacillus johnsonii* and *Lactobacillus plantarum* (LP), was reduced.

### The fecal microbiota transplantation of IS-treated mice caused anxiety in mice

**Figure 3:**
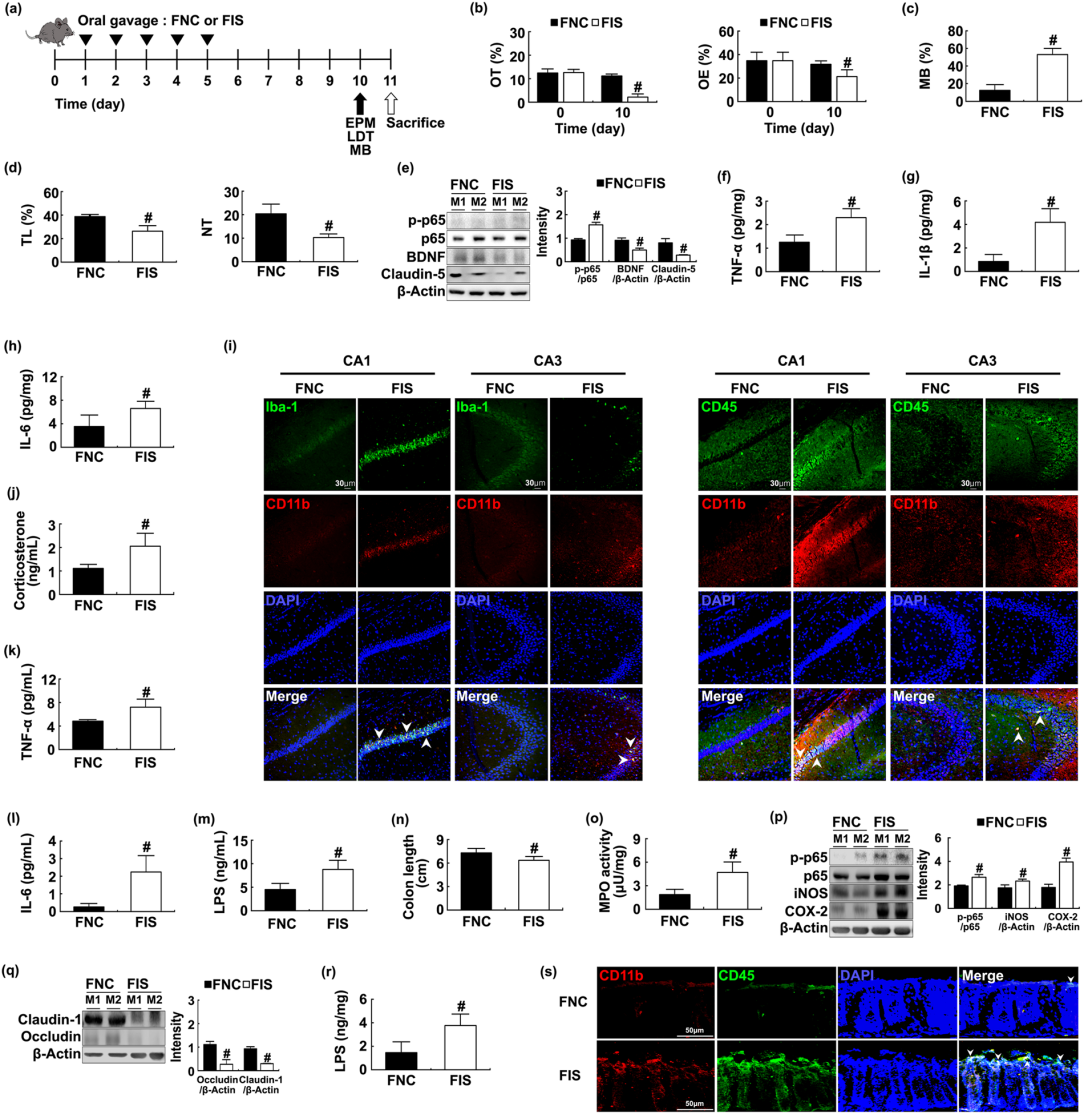
The fecal microbiota transplantation of IS-treated mice (FIS) induced anxiety-like behaviors and colitis in mice. FNC (fecal microbiota of control mice not treated with IS [NC]) and FIS were orally gavaged. Experimental time (**a**). Anxiety-like behaviors of mice were measured in the EPM (**b**, time spent in open arms [OT] and open arm entries [OE]), MB (marble buried, **c**) and LDT (**d**, time spent in light area [TL] and number of transitions [NT]) tasks. NF-κB (p-p65 and p65), BDNF, claudin-5, and β-actin were measured in the hippocampus by immunoblotting (**e**). Brain TNF-α (**f**) IL-1β (**g**) and IL-6 levels (**h**) were analyzed by ELISA kit. Blood corticosterone (**f**) IL-6 (**g**) and LPS levels (**h**) were analyzed by ELISA or LAL kit. Iba-1^+^/CD11b^+^ and CD11b^+^/CD11b^+^ populations were assayed by a confocal microscope (**i**). Blood corticosterone (**j**) TNF-α (**k**) IL-6 (**l**) and LPS levels (**m**) were analyzed by ELISA or LAL kit. Colitis markers colon length (**n**) and myeloperoxidase (MPO) activity (**o**) were measured in the colon. Colonic iNOS, COX-2, NF-κB (**p**) and tight junction proteins (occludin and claudin-1), and β-actin (**q**) were analyzed by immunoblotting. Fecal LPS level (**r**) and CD11b^+^/CD11b^+^ population (**s**) were analyzed by ELISA and confocal microscopy (**i**) respectively. FNC and FIS in figures indicate groups treated with fecal microbiota of control and IS-treated mice (suspended in 1% dextrose), respectively. All data except immunoblotting data (n = 4) were expressed as mean ± SD (n = 8). ^#^*p* < 0.05 vs. FNC group.

To confirm whether IS-induced anxiety was caused due to gut microbiota disturbance, we gavaged the fecal microbiota of IS-treated mice (FIS) to SPF mice and measured anxiety-like behaviors (Fig. 3a–e). Treatment with FIS significantly increased anxiety-like behaviors [F(1,14) = 320.35, *p* < 0.001; and F(1,14) = 21.491, *p* < 0.001 in OT and OE of EPM, respectively]; increased hippocampal NF-κB activation; suppressed hippocampal BDNF and claudin-5 expression; increased blood corticosterone [F(1,14) = 28.813, *p* < 0.001], IL-6 [F(1,14) = 49.754, *p* < 0.001], TNF-α [F(1,14) = 34.965, *p* < 0.001], and LPS levels [F(1,14) = 42.416, *p* < 0.001]; and induced the activated microglia/monocyte populations into the hippocampus (Figs 3e–i and S2a). The stimulation of FIS accumulated activated microglia (Iba^+^) and monocytes (CD11b^+^/CD45^+^) in the CA1 rather than the CA3 region of the hippocampus. Furthermore, FIS treatment increased corticosterone, IL-6, TNF-α, and LPS levels in the blood (Figs 3j–m and S2b). In contrast, no effect was observed on these levels in the mice treated with the fecal microbiota from SPF mice. Treatment with FIS also induced myeloperoxidase activity [F(1,14) = 99.536, *p* < 0.001]. FIS treatment also induced NF-κB activation as well as COX-2, iNOS, IL-6, TNF-α, and IL-1β expression. FIS treatment increased CD11b^+^/CD45^+^ population in the colon but suppressed the expression of tight junction proteins and IL-10 (Figs 3n–q,s and S2c–h). Treatment with FIS significantly increased the Proteobacteria population [F(1,14) = 17.385, *p* = 0.001] and fecal LPS levels [F(1,14) = 63.187, *p* < 0.001] (Figs 3r and S2i).

### Commensal bacteria *E*. *coli* and its LPS caused anxiety in mice

**Figure 4:**
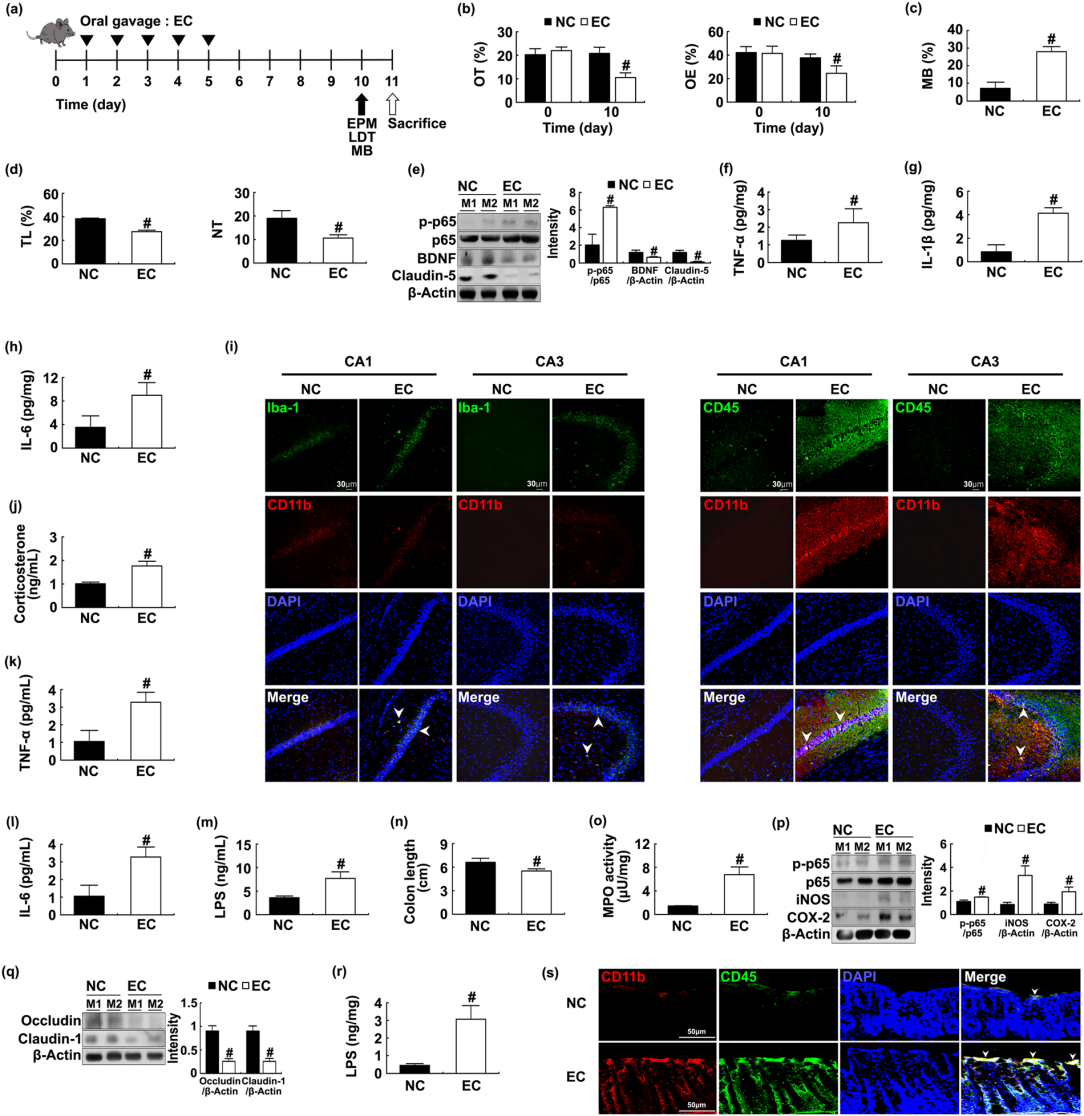
*Escherichia coli* caused anxiety and colitis in mice. *E*. *coli* (1 × 10^9^ CFU/0.2 mL/mouse) was orally gavaged. Experimental time (**a**). Anxiety-like behaviors of mice were measured in the EPM (**b**, time spent in open arms [OT] and open arm entries [OE]), MB (marble buried, **c**) and LDT (**d**, time spent in light area [TL] and number of transitions [NT]) tasks. NF-κB (p-p65 and p65), BDNF, claudin-5, and β-actin were measured in the hippocampus by immunoblotting (**e**). Brain TNF-α (**f**) IL-1β (**g**) and IL-6 levels (**h**) were analyzed by ELISA kit. Blood corticosterone (**f**) IL-6 (**g**) and LPS levels (**h**) were analyzed by ELISA or LAL kit. Iba-1^+^/CD11b^+^ and CD11b^+^/CD11b^+^ populations were assayed by a confocal microscope (**i**). Blood corticosterone (**j**) TNF-α (**k**) IL-6 (**l**) and LPS levels (**m**) were analyzed by ELISA or LAL kit. Colitis markers colon length (**n**) and myeloperoxidase (MPO) activity (**o**) were measured in the colon. Colonic iNOS, COX-2, NF-κB (**p**) and tight junction proteins (occludin and claudin-1), and β-actin (**q**) were analyzed by immunoblotting. Fecal LPS level (**r**) and CD11b^+^/CD11b^+^ population (**s**) were analyzed by ELISA and confocal microscopy (**i**) respectively. NC and EC in figures indicate groups treated with vehicle alone (1% dextrose) and *E*. *coli*, respectively. All data except immunoblotting data (n = 4) were expressed as mean ± SD (n = 8). ^#^*p* < 0.05 vs. NC group.

To understand whether IS-induced commensal bacteria could cause anxiety, we investigated the effect of *E*. *coli* on the anxiety occurrence in mice (Fig. 4). Oral administration of *E*. *coli* increased anxiety-like behaviors in the EPM [OT: F(1,14) = 195.270, *p* < 0.001; OE, F(1,14) = 72.269, *p* < 0.001], MB [F(1,14) = 310.116, *p* < 0.001], and LDT tasks [TL: F(1,14) = 898.780, *p* < 0.001; NT: F(1,14) = 81.038, *p* < 0.001] (Fig. 4a–d). Treatment with *E*. *coli* induced NF-κB activation and microglia/monocyte populations; suppressed BDNF and claudin-5 expression; and increased IL-6 [F(1,14) = 247.217, *p* < 0.001], TNF-α [F(1,14) = 11.542, *p* = 0.004], and IL-1β levels [F(1,14) = 154.991, *p* < 0.001] in the hippocampus (Figs 4e–i and S3a). *E*. *coli*-induced Iba+ and CD11b/CD45 (microglia/monocyte) populations were accumulated in the CA1 rather than in the CA3 region of hippocampus. However, almost no Iba+ and CD11b/CD45 populations were observed in the cortex of brain. Treatment with *E*. *coli* also increased corticosterone [F(1,14) = 264.831, *p* < 0.001], IL-6 [F(1,14) = 3848.148, *p* < 0.001], TNF-α [F(1,14) = 307.672, *p* < 0.001], IL-1β [F(1,14) = 966.156, *p* < 0.001], and LPS levels [F(1,14) = 46.084, *p* < 0.001] in the blood (Figs 4j–m and S3b). Moreover, *E*. *coli* treatment caused colitis: it caused colon shortening [F(1,14) = 73.263, *p* < 0.001]; increased myeloperoxidase activity [F(1,14) = 524.384, *p* < 0.001], IL-6 [F(1,14) = 10.814, *p* = 0.005], and TNF-α [F(1,14) = 57.048, *p* < 0.001] expression and monocyte population; suppressed the expression of tight junction proteins; increased NF-κB activation and CD11b^+^/CD45^+^ population in the colon; and increased Proteobacteria in gut microbiota (Figs 4n–r and S3c–i).

**Figure 5:**
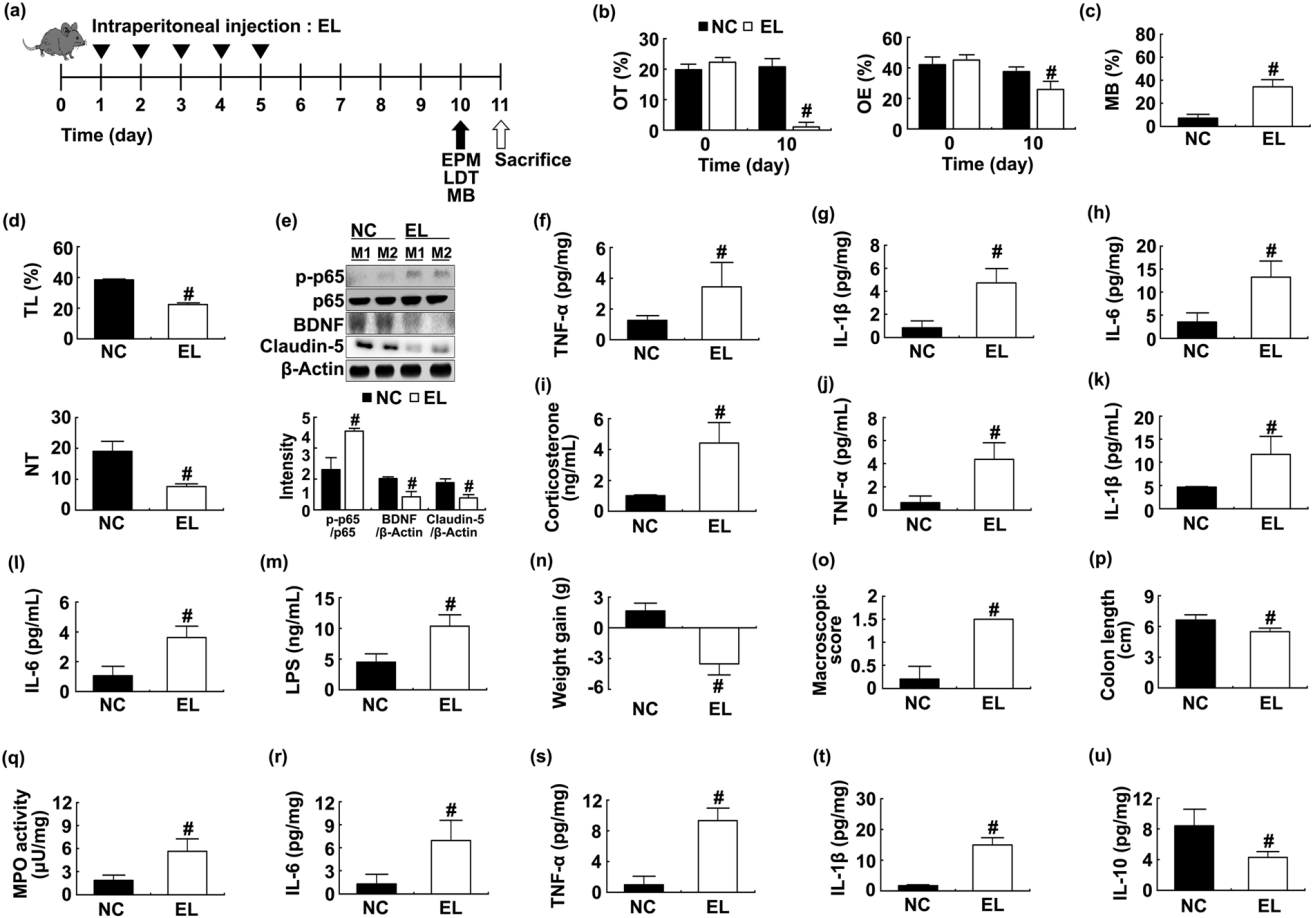
LPS purified from *Escherichia coli* (EL) caused anxiety and colitis in mice. Experimental schedule (**a**). Anxiety-like behaviors were measured on the 10^th^ day after the initial treatment with LPS in the EPM (**b**) MB (**d**) and LDT (**d**) tasks. LPS (10 μg/kg) was intraperitoneally treated for 5 days. NF-κB activation and BDNF and claudin-5 expression were measured in the hippocampus (**e**). Brain TNF-α (**f**) IL-1β (**g**) and IL-6 (**h**) levels were analyzed by ELISA kits. Blool corticosterone (**i**) IL-6 (**f**) TNF-α (**j**) IL-1β (**k**) IL-6 (**l**) and LPS (**m**) levels were by ELISA kits. Colitis markers body weight gain (**n**) macroscopic score (**o**) colon shortening (**p**) and MPO activity (**q**) IL-6 (**r**) TNF-α (**s**) IL-1β (**t**) and IL-10 (**u**) expression were measured in the colon. NC and EL in figures indicate groups treated with vehicle alone (1% dextrose) and *E*. *coli* LPS, respectively. All data except immunoblotting data (n = 4) were expressed as mean ± SD (n = 8). ^#^*p* < 0.05 vs. NC group.

Next, to understand whether the endotoxin(s) of *E*. *coli* could cause anxiety, we purified LPS from *E*. *coli* (EL) and investigated the effects of EL on the occurrence of anxiety and colitis in mice. The intraperitoneal injection of EL caused anxiety [OT: F(1,14) = 810.640, *p* < 0.001; OE, F(1,14) = 63.645, *p* < 0.001] (Figs 5a–d and S4). EL treatment induced NF-κB activation and IL-6, TNF-α, and IL-1β expression and suppressed BDNF and claudin-5 expression in the hippocampus (Fig. 5e–h). Treatment with EL also increased corticosterone [F(1,14) = 141.294, *p* < 0.001], IL-6 [F(1,14) = 294.258, *p* < 0.001], TNF-α [F(1,14) = 109.471, *p* < 0.001], and LPS levels [F(1,14) = 115,064, *p* < 0.001] in the blood (Fig. 5i–m). Treatment with EL also caused colitis; it increased colon shortening [F(1,14) = 52.600, *p* < 0.001] and myeloperoxidase activity [F(1,14) = 120.126, *p* = 0.001] and increased TNF-α [F(1,14) = 284.786, *p* < 0.001], IL-1β [F(1,14) = 818.012, *p* < 0.001], and IL-6 [F(1,14) = 72.483, *p* < 0.001] expression in the blood (Fig. 5n–u).

### *Lactobacilli* attenuated IS- and *E*. *coli*-induced anxiety in mice

**Figure 6:**
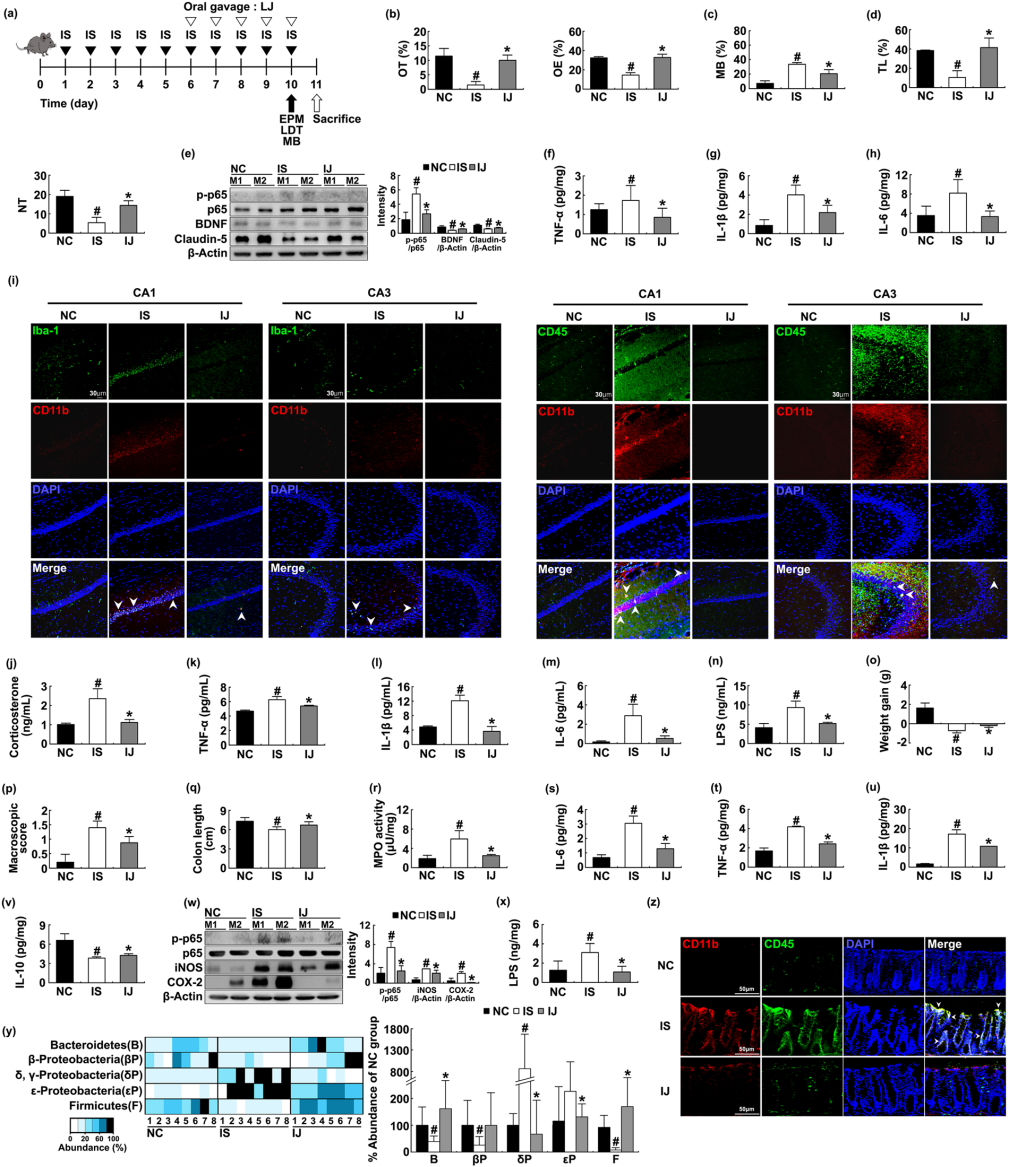
Oral administration of *Lactobacillus johnsonii* alleviated immobilization stress (IS)-induced anxiety-like behaviors and colitis in mice. Experimental schedule (**a**). Anxiety-like behaviors were measured on the 10^th^ day after the initial treatment with FIS in the EPM (**b**) MB (**c**) and LDT (**d**) tasks. IS was treated for 10 days. NF-κB activation and BDNF and claudin-5 expression were measured in the hippocampus (**e**). Brain TNF-α (**f**) IL-1β (**g**) and IL-6 (**h**) levels were analyzed by ELISA kits. Iba-1^+^/CD11b^+^ and CD11b^+^/CD11b^+^ populations were assayed in CA1 and CA3 regions of the hippocampus by a confocal microscope (**i**). Blood corticosterone (**j**) TNF-α (**k**) IL-1β (**l**) IL-6 (**m**) and LPS (**n**) levels in the blood. Colitis markers body weight gain (**o**) macroscopic score (**p**) colon shortening (**q**) and MPO activity (**r**) TNF-α (**s**) IL-1β (**t**) IL-6 (**u**) IL-10 (**v**), and NF-κB activation, iNOS, and COX-2 expression (**w**) were measured in the colon. Fecal LPS level (**x**) and gut microbiota (**y**) were measured in the feces. CD11b^+^/CD11b^+^ populations were assayed in the colon by a confocal microscope (**z**). NC, IS, and IJ in figures indicate groups treated with vehicle alone (1% dextrose) in normal control mice, vehicle in IS-treated mice, and *L*. *johnsonii* in IS-treated mice, respectively. The bacterial abundance was indicated as % of NC. All data except immunoblotting data (n = 4) were expressed as mean ± SD (n = 8). ^#^*p* < 0.05 vs. NC group. *p < 0.05 vs. IS-treated control (IS) group.

**Figure 7:**
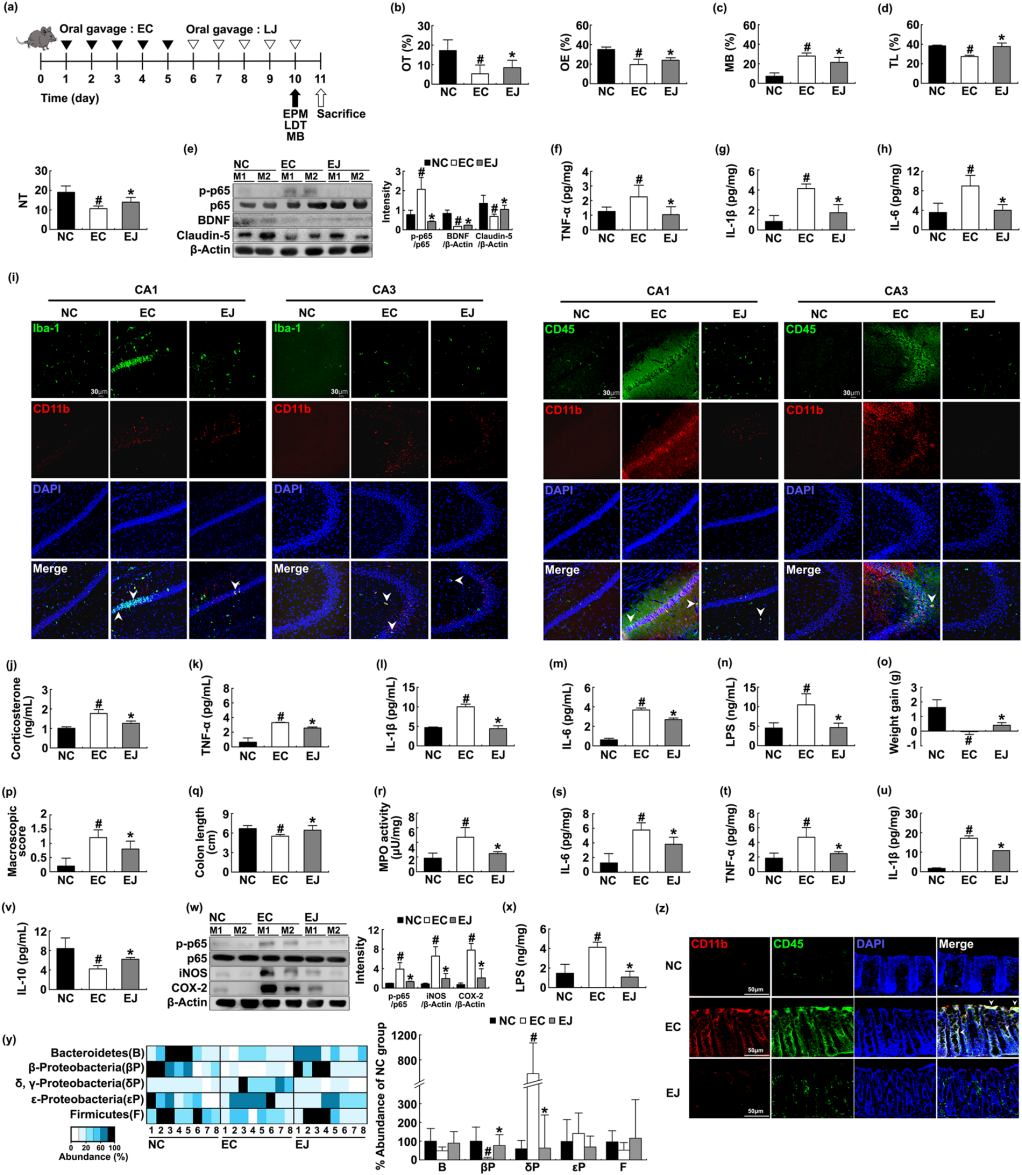
Oral administration of *Lactobacillus johnsonii* alleviated *Escherichia coli*-induced anxiety-like behaviors and colitis in mice. Experimental schedule (**a**). Anxiety-like behaviors were measured on the 10^th^ day after the initial treatment with FIS in the EPM (**b**) MB (**c**) and LDT (**d**) tasks. IS was treated for 10 days. NF-κB activation and BDNF and claudin-5 expression were measured in the hippocampus (**e**). Brain TNF-α (**f**) IL-1β (**g**) and IL-6 (**h**) levels were analyzed by ELISA kits. Iba-1^+^/CD11b^+^ and CD11b^+^/CD11b^+^ populations were assayed in CA1 and CA3 regions of the hippocampus by a confocal microscope (**i**). Blood corticosterone (**j**) TNF-α (**k**) IL-1β (**l**) IL-6 (**m**) and LPS (**n**) levels in the blood. Colitis markers body weight gain (**o**) macroscopic score (**p**) colon shortening (**q**) and MPO activity (**r**) TNF-α (**s**) IL-1β (**t**) IL-6 (**u**) IL-10 (**v**) and NF-κB activation, iNOS, and COX-2 expression (**w**) were measured in the colon. Fecal LPS level (**x**) and gut microbiota (**y**) were measured in the feces. CD11b^+^/CD11b^+^ populations were assayed in the colon by a confocal microscope (**z**). NC, EC, and EJ in figures indicate groups treated with vehicle alone (1% dextrose) in control mice, vehicle in *E*. *coli*-treated mice, and *L*. *johnsonii* in *E*. *coli*-treated mice, respectively. The bacterial abundance was indicated as % of NC. All data except immunoblotting data (n = 4) were expressed as mean ± SD (n = 8). ^#^*p* < 0.05 vs. NC group. *p < 0.05 vs. EC-treated control (EC) group.

To understand the effects of commensal *Lactobacilli* and *Bifidobacteria* on the occurrence of anxiety, we isolated *lactobacilli* including *L*. *johnsonii* and *L*. *plantarum* and investigated their effects against IS-induced anxiety in mice (Supplement Fig. S5). Oral gavage of *L*. *johnsonii* attenuated IS-induced anxiety-like behaviors more potently than *L*. *plantarum* treatment [OT: F(1,14) = 300.710, *p* < 0.001; OE, F(1,14) = 411.568, *p* < 0.001]; increased BDNF expression; and suppressed NF-κB activation in the hippocampus (Figs 6a–i and S4a). Exposure to IS increased TNF-α, IL-6, and LPS levels; and induced the infiltration of Iba^+^ and CD11b^+^/CD45^+^ population into the hippocampus, particularly CA3 region, while oral administration of *L*. *johnsonii* inhibited their infiltrations into the whole hippocampus. *L*. *johnsonii* treatment suppressed the infiltration of Iba^+^ and CD11b^+^/CD45^+^ populations into the hippocampus, particularly CA3 region. Treatment with IS increased corticosterone, TNF-α, IL-1β, IL-6, and LPS levels in the blood while *L*. *johnsonii* inhibited IS-induced corticosterone [F(1,14) = 97.820, *p* < 0.001], TNF-α [F(1,14) = 57.120, *p* < 0.001], IL-1β [F(1,14) = 296.441, *p* < 0.001), IL-6 [F(1,14) = 54.709, *p* < 0.001], and LPS levels [F(1,14) = 114.792, *p* < 0.001] in the blood (Fig. 6j–m). Moreover, treatment with *L*. *johnsonii* also alleviated IS-induced colitis; it suppressed colon shortening [F(1,14) = 15.561, *p* = 0.001], myeloperoxidase activity [F(1,14) = 82.048, *p* < 0.001], NF-κB activaton, and IL-6, TNF-α, IL-1β, iNOS, and COX-2 expression (Figs 6o–w and S4a–c). Treatment with *L*. *johnsonii* treatment reduced IS-induced gut microbiota LPS production, Proteobacteria population, and infiltration of CD11b^+^/CD45^+^ population into the colon (Fig. 6x–z). Moreover, *L*. *johnsonii* treatment attenuated *E*. *coli*-induced anxiety and colitis in mice, as in IS-treated mice (Fig. 7). *E*. *coli* treatment induced the infiltration of Iba^+^ and CD11b^+^/CD45^+^ populations into the hippocampus, particularly CA1 region, while oral administration of *L*. *johnsonii* inhibited their infiltrations into the whole hippocampus.

## Discussion

Gut microbiota are closely associated with the occurrence of obesity, autism, chronic inflammatory diseases, and asthma^34^. Recently, many studies have highlighted the role of gut microbiota on the outbreak of neuropsychiatric disorders via the MGB axis^9^. Upon exposure to stressors, germ-free mice displayed more exaggerated anxiety-like behaviors than did conventional ones. However, the transplantation of conventional mouse fecal microbiota into germ-free mice attenuated the hyperactive anxiety and reduced corticosterone levels in germ-free mice^15,29^. Exposure of conventional mice to stressors decreased *Clostridium* sp. and *Bacteroides* sp. populations in the gut microbiota and increased inflammatory cytokine expression and neuroendocrine hormone levels^32,35^. Furthermore, exposure to stressors can increase GI inflammation and intestinal permeability^36^, which can accelerate the translocation of gut bacteria and their byproducts such as LPS across the intestinal mucosa and directly stimulate both the immune and neuronal cells of ENS^37^. These findings support the MGB axis, suggesting that gut microbiota are closely associated with the outbreak of psychiatric disorders including anxiety. Nevertheless, the roles of commensal gut bacteria in the occurrence of anxiety disorders have been thoroughly studied.

In the present study, IS exposure increased anxiety-like behaviors, decreased hippocampal BDNF expression, induced hippocampal NF-κB activation, and increased activated microglia/monocytes populations in the hippocampus, particularly the CA3 region. Furthermore, IS exposure induced NF-κB activation in the colon and increased the filtration of monocyte population into the GI tract. Exposure of mice to IS also caused gut microbiota disturbance; it increased the Proteobacteria population while, like previously reported^32^, the Bacteroidetes population was decreased. Particularly, IS treatment increased the population of *Enterobacteriaceae* including *E*. *coli*, belonging to phylum Proteobacteria, and reduced the population of *Bifidobacteria* and *Lactobacilli* including *L*. *johnsonii*. Exposure to IS increased fecal and blood LPS levels and suppressed tight junction protein expression in the brain and colon. These findings suggest that exposure to IS can cause GI inflammation and anxiety via HPA axis. Moreover, the IS-induced GI inflammation may accelerate the absorption of the gut microbiota LPS into the blood and brain through the GI membrane, like previously reported in mice with 2,4,6-trinitrobenzenesulfonic acid-induced colitis^38^. This may be because exposure to IS can induce gut bacterial LPS production through the gut microbiota disturbance, leading to the GI inflammation. IS exposure also suppressed hippocampal claudin-1, claudin-5, and occludin expression and increased hippocampal NF-κB activation. These findings suggest that IS exposure is able to accelerate LPS transport into the brain by damaging brain-blood barrier function, resulting in neuroinflammation. Oral administration of FIS or *E*. *coli* caused gut microbiota disturbance and GI inflammation; these treatments increased TNF-α expression and NF-κB activation, suppressed tight junction protein expression in the colon, and increased fecal LPS levels. These treatments also increased anxiety-like behaviors and suppressed hippocampal CREB phosphorylation and BDNF expression. Moreover, treatment with FIS or *E*. *coli* stimulated the infiltration of activated microglia (Iba1^+^) and monocytes (CD11b^+^/CD45^+^) into the hippocampal CA1 and CA3 regions. Particularly, activated microglia and monocytes were accumulated in the CA1 rather than in the CA3 region of mice treated with FIS or *E*. *coli* while these cells were in the CA3 rather than the CA1 region of the hippocampus of IS-exposed mice. Moreover, oral administration of *L*. *johnsonii* inhibited the infiltration of activated microglia and monocytes into the whole hippocampus by the stimulation of *E*. *coli* or IS. These findings suggest that the disturbance of gut microbiota composition by exposure to IS may increase fecal and blood LPS levels, activate systemic monocytes, successively activate microglia in the entire hippocampus via the CA1 region, and suppress BDNF expression via the activation of NF-κB in the brain, resulting in anxiety.

Thus, the excessive proliferation of *E*. *coli* by stresses may deteriorate anxiety through MGB axis. Additionally, *L*. *johnsonii*, one of commensal *lactobacilli* in mice, alleviated IS- or *E*. *coli*-induced anxiety-like behaviors as well as colitis. *L*. *johnsonii* also restored the gut microbiota disturbance and fecal LPS levels. These results suggest that commensal *lactobacilli* including *L*. *johnsonii* may mitigate anxiety through the suppression of GI inflammation by restoring the gut microbiota disturbance and inhibiting excessive gut bacterial LPS production. Additionally, *Lactobacillus plantarum* attenuated anxiety and gut microbiota disturbance^39^. *Bifidobacterium longum* NCC3001 attenuated anxiety-like behaviors in rats^40^. *Bifidobacterium infantis* treatment reduced depressive-like behavior and restored noradrenaline levels in the brainstems of rats^41^. *Bifidobacteria* and *Lactobacilli* attenuated anxiety- and depression-like behaviors by inducing GABA production^42^. Moreover, probiotics exhibit a variety of gut membrane-mediated biological activities such as inhibition of bacterial translocation^43^, enhancement of mucosal barrier function^44^, induction of colonic AH neuron excitability^45^, and induction of cannabinoid and opioid receptors in intestinal epithelial cells^46^. In the present study, we found that *L*. *johnsonii*, a commensal gut bacterium, suppressed IS- or *E*. *coli*-induced gut microbiota LPS production and induced IS- or *E*. *coli*-suppressed gut tight junction protein expression in mice. These results suggest that *Lactobacilli* and *Bifidobacteria*, which are beneficial commensal bacteria, can relieve both anxiety and colitis by inhibiting NF-κB activation and inducing BDNF expression; these effects can be alleviated by correcting gut microbiota disturbance. Furthermore, gut microbiota composition as well as intestinal permeability play important roles in the occurrence of psychiatric disorders. These results support the hypothesis that the relationship between gut microbiota and psychiatric disorders is bidirectional^47^; stressors can disturb the gut microbial composition through the release of stress hormones or immune cytokines and gut microbiota disturbed by IS can cause anxiety by inducing gut neuroinflammation and gut inflammation.

In conclusion, the induction of Proteobacteria populations, particularly *E*. *coli*, and their LPS production can increase the occurrence of anxiety through GI inflammation while the restoration of stress-disturbed gut microbiota composition by treatment with beneficial bacteria may be helpful in alleviating anxiety through the amelioration of GI inflammation.

## Materials and Methods

### Materials

Enzyme-linked immunosorbent assay (ELISA) kits for corticosterone and cytokines were purchased from Ebioscience (San Diego, CA). 4′,6-Diamidino-2-phenylindole dilactate (DAPI) was purchased from Sigma (St. Louis, MO). Antibodies for p65, p-p65, BDNF, claudin-5, occludin, caludin-1, iNOS, COX-2, and β-actin were purchased from Cell Signaling Technology (Beverly, MA). QIAamp Fast DNA stool mini kit was purchased from Qiagen (Hilden, Germany). Limulus amoebocyte lysate (LAL) assays was purchased from Cape Cod Inc. (East Falmouth, MA). Hydrogen sulfate lactose medium (DHL) was purchased from Eiken Chem (Tokyo, Japan) and general anaerobic medium (GAM) and Blood liver medium (BL) were from Nissui Pharmaceutical Co. (Tokyo, Japan). MRS medium was purchased from BD (Radnor, PA).

### Culture of gut bacteria

Gut bacteria were cultured by serially diluting fresh mouse feces (approximately 0.1 g) with GAM broth and inoculating onto selective agar plates, such as DHL and BL agar plates, according to the method of Jang *et al*.^38^. The DHL agar plates were aerobically cultured at 37 °C for 1 day while anaerobically culturing the BL agar plates for 3 days under the same condition.

*Lactobacilli* (*L*. *johnsonii* and *L*. *plantarum*) and *E*. *coli* isolated from mouse feces were cultured in GAM broth and Gram staining, 16S rRNA sequencing, and API 50 CHL kit for a sugar utilization test were conducted to identify isolated bacteria. The characteristics of *E*. *coli* are shown in Supplement Fig. S6. In short, the isolated fecal bacteria were anaerobically cultivated to the density of 0.5–0.8 at 600 nm in GAM broth (0.3 L) at 37 °C, collected through centrifugation at 5,000 *g* for 20 min, and washed twice with saline. Thereafter, the collected cells (5 × 10^9^ CFU/mL) were suspended in 1% glucose for *in vivo* experiments.

### Animals

Male C57BL/6 mice (6-weeks-old, 20~22 g) were bought from Orient Animal Breeding Center (Seoul, Korea) and housed in wire cages (four mice per cage) under controlled conditions where the temperature was maintained at 24 ± 2 °C and humidity at 60 ± 10% with an alternating light-dark cycle (12 h/12 h). The mice were provided with standard laboratory rodent chow and tap water ad libitum. All animal experiments were approved by the Institutional Animal Care and Use Committee of the Kyung Hee University (IRB Number: KUASP(SE)-16011) and performed according to the NIH and University Guide for Laboratory Animals Care and Usage.

### Generation of mice with anxiety

Mice were acclimated for one week before experiments. Mice with anxiety were prepared by treatment with IS or gavage of the fecal microbiota of mice treated with IS (FIS) or not treated with IS (FNC), *E*. *coli*, or *E*. *coli* LPS (EL). Each group contained eight mice.

First, to generate mice with IS-induced anxiety, each mouse was inserted into a 35-mL conical tube-like instrument (2.5 cm in diameter, 7.5 cm in length) with a 0.25-cm-diameter hole on the center of the tube, stuffed to prevent forward-to-backward and side-to-side mobility, and vertically placed for 2 h^48^. IS was instituted once daily for 10 days. The anxiety-like behaviors were assessed 24 h before treatment with IS and on the 5^th^ and 10^th^ days after IS treatment. Second, mice were orally gavaged with FNC or FIS (0.2 mL suspended in 1% dextrose) once daily for 5 days, and mouse behaviors were assessed on the 5^th^ day after the final gavage of FNC or FIS. The preparation of FIS and FNC was as follows: the fresh feces (1 g) of mice treated with IS or not treated with IS were gathered 24 h after the final treatment with IS, suspended in GAM broth (9 mL) on ice, centrifuged at 500 *g* and 4 °C for 5 min, washed with 1% dextrose twice, and suspended in 1% dextrose. The suspended fecal microbiota (20 mg of feces/0.2 mL/mouse) were orally gavaged in mice. Third, mice were orally gavaged with *E*. *coli* (1 × 10^9^ CFU/0.2 mL/mouse/day) once daily for 5 days. Mouse behaviors were assessed on the 4^th^ day after the final treatment with *E*. *coli*. Fourth, EL (5, 10, or 50 μg/kg, dissolved in saline [0.1 mL/mouse]) was intraperitoneally injected in mice once daily for 5 days. Mouse behaviors were assessed on the 4^th^ day after the final treatment of EL. EL was purified according to the method of Jang *et al*.^38^.

To investigate the effects of *Lactobacilli* against anxiety, mice were divided into four groups treated with IS-untreated (control), IS alone, *L*. *johnsonii* with IS, and *L*. *plantarum* with IS. Mice were vertically placed in conical tube-like instrument for 2 h once a day and treated with IS for 10 days. *L*. *johnsonii* (1 × 10^9^ CFU/mouse/day, *p*.*o*.) and *L*. *plantarum* (1 × 10^9^ CFU/mouse/say, *p*.*o*.) were orally administered from the 6^th^ day to 10^th^ day from the initial IS treatment. Next, to evaluate the ameliorating effects of *L*. *johnsonii* against *E*. *coli*-induced anxiety, mice were randomly divided into three groups treated with *E*. *coli*-untreated (control), *E*. *coli* alone, and *L*. *johnsonii* (1 × 10^9^ CFU/mouse/day, *p*.*o*.) with *E*. *coli*. *E*. *coli* (1 × 10^9^ CFU/mouse/day) was orally gavaged once a day for 5 days. *L*. *johnsonii* (1 × 10^9^ CFU/mouse/day) was orally administered from the next day to 5^th^ day after the final *E*. *coli* treatment. Control group was treated with saline instead of *E*. *coli*. Control, IS-treated, and *E*. *coli*-treated groups were gavaged with vehicle (1% dextrose) instead of test agents. Anxiety-like behaviors were measured 8 h after the final *lactobacilli* treatment. Mice were sacrificed 12 h after the final *lactobacilli* treatment.

### Behavioral tasks

EPM task was assessed in the plus-maze apparatus according to the method of Oh *et al*.^48^. The maze had two open (30 × 7 × 1 cm) and two enclosed arms (30 × 7 × 20 cm, each) extending from a central platform (7 × 7 cm) elevated to a height of 50 cm above the floor. LDT task was assessed in the light/dark box apparatus (45 × 25 × 25 cm) according to the method of Jindal *et al*.^49^. This maze had two chambers with black and white polywood walls and Plexiglass for floors. These chambers were connected by an opening (7.5 × 7.5 cm) located in the center of the dividing wall at floor level. MB task was assessed in a cage (30 × 36 × 13 cm) according to the method of Savignac *et al*.^50^. This maze is made of smooth, opaque plastic with a 5-cm layer of sawdust.

### Immunoblotting

The mice were sacrificed 2 h after performing the final behavioral tasks. Thereafter, their hippocampal and colon tissues were lysed with ice-cold lysis RIPA buffer containing 50 mM Tris–HCl (pH 8.0), 1% phosphatase inhibitor cocktail and a protease inhibitor cocktail, 150 mM sodium chloride, 0.5% sodium deoxycholate, 1.0% Igepal CA-630 (NP-40), and 0.1% sodium dodecyl sulfate (SDS)^38^. The lysates were centrifuged at 10,000 *g* and 4 °C for 10 min. The resulting supernatants were electrophoresed by SDS-polyacrylamide gel electrophoresis, transferred to a nitrocellulose membrane using Western Blot Transfer Sysem (Bio Rad Laboratories, Inc, Hercules, CA), blocked with 5% non-fat dried-milk proteins, probed with the antibodies for p65, p-p65, BDNF, claudin-5, occludin, caludin-1, iNOS, COX-2, and β-actin, and washed twice with phosphate buffered saline with tween 20. The membranes were incubated with horseradish peroxidase-conjugated secondary antibodies. The protein bands were detected by using enhanced chemiluminescence detection kit.

### ELISA assay

The bloods collected from carotid artery were centrifuged at 3000 *g*, 4 °C for 5 min for the assay of corticosterone and cytokines in the blood. The resulting sera were prepared^48,51^. Levels of corticosterone, IL-6, TNF-α, and IL-1β in the sera and tissue homogenate supernatants were assessed using ELISA kits.

### Immunofluorescence assay

The mice were trans-cardiacally perfused with 4% paraformaldehyde for brain and colon tissues fixation. Brains and colons were post-fixed with 4% paraformaldehyde for 4 h, cytoprotected in 30% sucrose solution, freezed, and cut using a cryostat (Leica, Nussloch, Germany). Immunostaining was performed according to the method of Tronnes *et al*.^52^. CD45 (1:100, Abcam) and CD11b (1:150, Abcam) antibodies were stained to detect CD11b^+^ immune cells including monocytes. An Iba-1 antibody (1:100, Santa Cruz Biotechnology) was stained to detect microglial cells. In short, brains and colons were cryoprotected in 30% sucrose-phosphate buffered saline, frozen with optimal cutting temperature compound (Agar Scientific Ltd., Essex, UK), and stored at −80 °C until the usage of experiments. These blocked tissues were cryosectioned (thickness, 30 μm), stored at 4 °C in the storing solution (phosphate-buffered saline containing 30% glycerol and 30% ethylene glycol), permeabilized in 0.5% Triton X-100 for 5 min, and blocked in 10% bovine serum albumin with phosphate buffered saline with tween 20 for 30 min. Thereafter, the cryosectioned tissues were incubated for 16 h at 4 °C with the antibodies for Iba1, CD45, and CD11b and treated with secondary antibodies conjugated with Alexa Fluor 488 (1:1,000, Invitrogen) or Alexa Fluor 594 (1:500, Abcam) thereafter. The nuclei were stained with DAPI. The immunostained tissues were observed with a confocal laser microscope.

### Quantitative polymerase chain reaction (qPCR)

qPCR for gut microbiota was carried out with total DNA (0.1 μg), which was isolated from the feces, with SYBER premix in a Takara thermal cycler according to the method of Yang *et al*.^53^. The thermal cycling conditions were 95 °C for 30 s, followed by 35 cycles at 95 °C for 5 s (denaturation) and 63 °C for 30 s (annealing and extension). The bacterial population level was calculated relative to 16S ribosomal RNA by using Microsoft Excel. Abundance (%) indicates [the population of each phylum in each feces]/[the population of each phylum in the most highest one] × 100. Primers were used in Supplement Table S1.

### Pyrosequencing

Genomic DNA was isolated from the fresh feces of mice using a QIAamp Fast DNA stool mini kit according to the method of Jeong *et al*.^51^. PCR amplification was performed by using primers targeting the V3 to V4 regions of 16S ribosomal RNA genes with gut bacterial genomic DNA. For the amplification, barcode-containing fusion primers were used. The sequencing for equimolar concentration of each amplicon was carried out at Chunlab Inc. (Seoul, Korea) with Illumina MiSeq System, as stated in the manufacturer’s directions.

Reads taken from different samples were classified by unique barcodes of each PCR product and the target region in barcoded primers was identified. All of the linked sequences including adapter, barcode, and linker and low quality sequences (reads with two or more indefinite nucleotides, a low quality score, or <500 bp) were eliminated. Potential chimeric sequences were confirmed by the Bellerophon formula. The taxonomic sorting of each read was assigned against the EzTaxon-e database (http://eztaxon-e.ezbiocloud.net), which has the 16S rRNA gene sequence of type strains that have valid published names and repesentative species level phylotypes of either cultured or uncultured entries in the GenBank database with complete hierarchical taxonomic classification from phyla to species. 16S rRNA gene sequences originated from our study were deposited in NCBI’s SRA (SRX4051778~4051782, SRX3153173, SRX3153178~3153180, SRX3153182). The species richness of samples was determined using the CLcommunity program. Subsampling was randomly performed to equalize the read size of tested samples to compare the different read size within tested samples. For the comparison of the OTUs between tested samples, shared OTUs were obtained with the XOR analysis of the CLcommunity program.

### LAL assay

The contents of fecal and blood endotoxins were assessed using the diazo-coupled LAL assay kit^51,54^. In short, mouse fresh feces (20 mg) was gathered in a pyrogen-free tube, suspended in 30 mL of phosphate buffered saline, sonicated for 60 min on ice, and centrifuged (400 *g*, 15 min). The supernatant (20 mL) was collected, sequentially filtrated through a 0.45 µm and 0.22 µm filters. The filtrate was inactivated for 10 min at 70 °C. The endotoxin content was measured. For the assay of blood endotoxin levels, the sera were diluted in water 10-fold, heated at 70 °C for 10 min, and centrifuged at 3,000 *g* for 10 min. The endotoxin content was assayed.

### Statistical analysis

Experimental data were expressed as mean ± SD and statistically analyzed using one-way ANOVA followed by Duncan’s multiple range test (*p* < 0.05). All *p*-values were described in Supplement Table S2.

## Electronic supplementary material

Supplement
