# Supplementary material for: Immobilization stress-induced Escherichia coli causes anxiety by inducing NF-κB activation through gut microbiota disturbance
Source: Sci Rep. 2018 Sep 17;8:13897. doi: 10.1038/s41598-018-31764-0 (PMC6141499; doi:10.1038/s41598-018-31764-0)
Supplement: Supplementary file 1 — Supplement [file 41598_2018_31764_MOESM1_ESM.pdf]

[Supplement]

Immobilization stress-induced *Escherichia coli* causes anxiety by inducing NF-κB activation through gut microbiota disturbance

Hyo-Min Jang, Kyung-Eon Lee, Hae-Ji Lee, and Dong-Hyun Kim*

Neurobiota Research Center and Department of Life and Nanopharmaceutical Sciences, College of Pharmacy, Kyung Hee University, 26, Kyungheedae-ro Dongdaemun-gu, Seoul 02447, Korea

Running title: *Escherichia coli* caused anxiety

*Correspondence: Dong-Hyun Kim, Ph.D.

Department of Life and Nanopharmaceutical Sciences and Department of Pharmacy, College of Pharmacy, Kyung Hee University,

26, Kyungheedae-ro, Dongdaemun-gu, Seoul, 02447, Korea

Tel +82-2-961-0374

E-mail dhkim@khu.ac.kr

Supplement Table S1. Primers for quantitative polymerase chain reaction (qPCR)

Phylum	Primer sequence	
	Forward	Reverse
Firmicutes	5'-GGA GYA TGT GGT TTA ATT CGA AGC A-3'	5'-AGC TGA CGA CAA CCA TGC AC-3'
Bacteroidetes	5'-GTT TAA TTC GAT GAT ACG CGA G –3'	5'-TTA ASC CGA CAC CTC ACG G –3'
β-Proteobacteria	5'-AAC GCG AAA AAC CTT ACC TAC C-3'	5'-TGC CCT TTC GTA GCA ACT AGT G-3'
δ/γ-Proteobacteria	5'-GCT AAC GCA TTA AGT RYC CCG-3'	5'-GCC ATG CRG CAC CTG TCT-3'
ε-Proteobacteria	5'-TAG GCT TGA CAT TGA TAG AAT C-3'	5'-CTT ACG AAG GCA GTC TCC TTA-3'
Bacterial 16S rRNA	5'-TCG TCG GCA GCG TCA GAT GTG TAT AAG AGA CAG GTG CCA GCM GCC GCG GTA A-3'	5'-GTC TCG TGG GCT CGG AGA TGT GTA TAA GAG ACA GGG ACT ACH VGG GTW TCT AAT-3'

Supplement Table S2. *P*-values in all experiments

Figure 1			
NC&IS			
(b)	EPM	OT (%)	OE (%)
	0 day	F(1,14)=0.015 p=0.903	F(1,14)=0.000 p=1.000
	5th day	F(1,14)=24.295 p=0.000	F(1,14)=19.073 p=0.001
	10th day	F(1,14)=30.193 p=0.000	F(1,14)=22.847 p=0.000
(c)	MB (%)	F(1,14)=22.487 p=0.000	
(d)	LDT	TL (%)	NT
		F(1,14)=74.326 p=0.000	F(1,14)=51.368 p=0.000
(e)	p-p65/p65	F(1,6)=25.439 p=0.002	
	BDNF/β-Actin	F(1,6)=17.527 p=0.006	
	Claudin-5/β-Actin	F(1,6)=351.013 p=0.000	
(f)	TNF-α (pg/mg)	F(1,14)=5.099 p=0.040	
(g)	IL-1β (pg/mg)	F(1,14)=59.511 p=0.000	
(h)	IL-6 (pg/mg)	F(1,14)=14.867 p=0.002	
(j)	Corticosterone (ng/mL)	F(1,14)=32.620 p=0.000	
(k)	TNF-α (pg/mL)	F(1,14)=26.117 p=0.000	
(l)	IL-6 (pg/mL)	F(1,14)=28.467 p=0.000	
(m)	LPS (ng/mL)	F(1,14)=46.084 p=0.000	
(n)	Colon length (cm)	F(1,14)=19.243 p=0.001	
(o)	MPO activity (μU/mg)	F(1,14)=301.258 p=0.000	
(p)	p-p65/p65	F(1,6)=41.128 p=0.001	
	iNOS/β-Actin	F(1,6)=70.674 p=0.000	
	COX-2/β-Actin	F(1,6)=542.118 p=0.000	
(q)	Occludin/β-Actin	F(1,6)=94.855 p=0.000	
	Claudin-1/β-Actin	F(1,6)=100.045 p=0.000	

(r)	LPS (ng/mg)	F(1,14)=25.920 p=0.000	

Figure 2			
NC&IS			
(f)	BL	*Bif+Lac*	
	0 day	F(1,14)=0.015 p=0.903	
	10th day	F(1,14)=19.347 p=0.001	
(g)	DHL	*Enterobact*	*E. coli*
	0 day	F(1,14)=0.258 p=0.619	F(1,14)=0.119 p=0.736
	10th day	F(1,14)=99.046 p=0.000	F(1,14)=44.038 p=0.000

Figure 3			
FNC&FIS			
(b)	EPM	OT (%)	OE (%)
	0 day	F(1,14)=0.157 p=0.698	F(1,14)=0.000 p=1.000
	10th day	F(1,14)=320.35 p=0.000	F(1,14)=21.491 p=0.000
(c)	MB (%)	F(1,14)=168.538 p=0.000	
(d)	LDT	TL (%)	NT
		F(1,14)=57.364 p=0.000	F(1,14)=43.698 p=0.000
(e)	p-p65/p65	F(1,6)=125.311 p=0.000	
	BDNF/β-Actin	F(1,6)=48.775 p=0.000	
	Claudin-5/β-Actin	F(1,6)=31.932 p=0.001	
(f)	TNF-α (pg/mg)	F(1,14)=35.575 p=0.000	
(g)	IL-1β (pg/mg)	F(1,14)=52.690 p=0.000	
(h)	IL-6 (pg/mg)	F(1,14)=14.404 p=0.002	
(j)	Corticosterone (ng/mL)	F(1,14)=28.813 p=0.000	
(k)	TNF-α (pg/mL)	F(1,14)=34.965 p=0.000	
(l)	IL-6 (pg/mL)	F(1,14)=49.754 p=0.000	
(m)	LPS (ng/mL)	F(1,14)=42.416 p=0.000	
(n)	Colon length (cm)	F(1,14)=16.104 p=0.001	
(o)	MPO activity (μU/mg)	F(1,14)=99.536 p=0.000	
(p)	p-p65/p65	F(1,6)=37.136 p=0.001	
	iNOS/β-Actin	F(1,6)=16.306 p=0.007	
	COX-2/β-Actin	F(1,6)=111.654 p=0.000	
(q)	Occludin/β-Actin	F(1,6)=50.862 p=0.000	
	Claudin-1/β-Actin	F(1,6)=200.730 p=0.000	
(r)	LPS (ng/mg)	F(1,14)=63.187 p=0.000	

Figure 4			
NC&EC			
(b)	EPM	OT (%)	OE (%)
	0 day	F(1,14)=0.635 p=0.439	F(1,14)=0.048 p=0.829
	10th day	F(1,14)=195.27 p=0.000	F(1,14)=72.269 p=0.000
(c)	MB (%)	F(1,14)=310.116 p=0.000	
(d)	LDT	TL (%)	NT
		F(1,14)=898.78 p=0.000	F(1,14)=81.038 p=0.000
(e)	p-p65/p65	F(1,6)=48.356 p=0.000	
	BDNF/β-Actin	F(1,6)=20.158 p=0.004	
	Claudin-5/β-Actin	F(1,6)=83.009 p=0.000	
(f)	TNF-α (pg/mg)	F(1,14)=11.542 p=0.004	
(g)	IL-1β (pg/mg)	F(1,14)=154.991 p=0.000	
(h)	IL-6 (pg/mg)	F(1,14)=27.217 p=0.000	

(j)	Corticosterone (ng/mL)	F(1,14)=264.831 p=0.000	
(k)	TNF-α (pg/mL)	F(1,14)=307.672 p=0.000	
(l)	IL-6 (pg/mL)	F(1,14)=3848.148 p=0.000	
(m)	LPS (ng/mL)	F(1,14)=46.084 p=0.000	
(n)	Colon length (cm)	F(1,14)=73.263 p=0.000	
(o)	MPO activity (μU/mg)	F(1,14)=524.384 p=0.000	
(p)	p-p65/p65	F(1,6)=39.936 p=0.001	
	iNOS/ β-Actin	F(1,6)=34.845 p=0.001	
	COX-2/β-Actin	F(1,6)=26.318 p=0.002	
(q)	Occludin/β-Actin	F(1,6)=107.345 p=0.000	
	Claudin-1/β-Actin	F(1,6)=109.532 p=0.000	
(r)	LPS (ng/mg)	F(1,14)=493.576 p=0.000	

Figure 5			
NC&EL			
(b)	EPM	OT (%)	OE (%)
	0 day	F(1,14)=3.270 p=0.092	F(1,14)=0.907 p=0.357
	10th day	F(1,14)=810.64 p=0.000	F(1,14)=63.645 p=0.000
(c)	MB (%)	F(1,14)=217.581 p=0.000	
(d)	LDT	TL (%)	NT
		F(1,14)=2312.7 p=0.000	F(1,14)=157.296 p=0.000
(e)	p-p65/p65	F(1,6)=40.546 p=0.001	
	BDNF/β-Actin	F(1,6)=46.639 p=0.000	
	Claudin-5/β-Actin	F(1,6)=33.892 p=0.001	
(f)	TNF-α (pg/mg)	F(1,14)=14.955 p=0.002	
(g)	IL-1β (pg/mg)	F(1,14)=65.912 p=0.000	
(h)	IL-6 (pg/mg)	F(1,14)=47.552 p=0.000	
(i)	Corticosterone (ng/mL)	F(1,14)=141.294 p=0.000	
(j)	TNF-α (pg/mL)	F(1,14)=109.471 p=0.000	
(k)	IL-1β (pg/mL)	F(1,14)=46.204 p=0.000	
(l)	IL-6 (pg/mL)	F(1,14)=294.258 p=0.000	
(m)	LPS (ng/mL)	F(1,14)=115.064 p=0.000	
(n)	Weight gain (g)	F(1,14)=259.431 p=0.000	
(o)	Macroscopic score	F(1,14)=317.301 p=0.000	
(p)	Colon length (cm)	F(1,14)=52.600 p=0.000	
(q)	MPO activity (μU/mg)	F(1,14)=120.126 p=0.000	
(r)	IL-6 (pg/mg)	F(1,14)=72.483 p=0.000	
(s)	TNF-α (pg/mg)	F(1,14)=294.786 p=0.000	
(t)	IL-1β (pg/mg)	F(1,14)=818.012 p=0.000	
(u)	IL-10 (pg/mg)	F(1,14)=47.557 p=0.000	

Figure 6			
NC&IS, IS&IJ			
(b)	EPM	OT (%)	OE (%)
		F(1,14)=203.35 p=0.000<br>F(1,14)=300.71 p=0.000	F(1,14)=754.038 p=0.000<br>F(1,14)=411.568 p=0.000
(c)	MB (%)	F(1,14)=163.557 p=0.000<br>F(1,14)=15.839 p=0.001	
(d)	LDT	TL (%)	NT
		F(1,14)=74.326 p=0.000	F(1,14)=51.368 p=0.000

		F(1,14)=73.868 p=0.000	F(1,14)=71.502 p=0.000
(e)	p-p65/p65	F(1,6)=28.207 p=0.002<br>F(1,6)=29.117 p=0.002	
	BDNF/β-Actin	F(1,6)=28.571 p=0.002<br>F(1,6)=45.127 p=0.001	
	Claudin-5/β-Actin	F(1,6)=46.733 p=0.000<br>F(1,6)=7.948 p=0.030	
(f)	TNF-α (pg/mg)	F(1,14)=5.099 p=0.040<br>F(1,14)=7.421 p=0.016	
(g)	IL-1β (pg/mg)	F(1,14)=59.511 p=0.000<br>F(1,14)=17.225 p=0.001	
(h)	IL-6 (pg/mg)	F(1,14)=14.867 p=0.002<br>F(1,14)=20.718 p=0.000	
(j)	Corticosterone (ng/mL)	F(1,14)=123.194 p=0.000<br>F(1,14)=97.820 p=0.000	
(k)	TNF-α (pg/mL)	F(1,14)=191.865 p=0.000<br>F(1,14)=57.120 p=0.000	
(l)	IL-1β (pg/mL)	F(1,14)=355.129 p=0.000<br>F(1,14)=296.441 p=0.000	
(m)	IL-6 (pg/mL)	F(1,14)=77.534 p=0.000<br>F(1,14)=54.709 p=0.000	
(n)	LPS (ng/mL)	F(1,14)=128.427 p=0.000<br>F(1,14)=114.792 p=0.000	
(o)	Weight gain (g)	F(1,14)=122.327 p=0.000<br>F(1,14)=7.003 p=0.019	
(p)	Macroscopic score	F(1,14)=65.258 p=0.000<br>F(1,14)=9.000 p=0.010	
(q)	Colon length (cm)	F(1,14)=29.878 p=0.000<br>F(1,14)=15.561 p=0.001	
(r)	MPO activity (μU/mg)	F(1,14)=108.075 p=0.000<br>F(1,14)=82.048 p=0.000	
(s)	IL-6 (pg/mg)	F(1,14)=414.934 p=0.000<br>F(1,14)=203.073 p=0.000	
(t)	TNF-α (pg/mg)	F(1,14)=1114.055 p=0.000<br>F(1,14)=3116.768 p=0.000	
(u)	IL-1β (pg/mg)	F(1,14)=1168.241 p=0.000<br>F(1,14)=187.951 p=0.000	
(v)	IL-10 (pg/mg)	F(1,14)=202.862 p=0.000<br>F(1,14)=39.217 p=0.000	
(w)	p-p65/p65	F(1,6)=38.153 p=0.001<br>F(1,6)=35.633 p=0.001	
	iNOS/β-Actin	F(1,6)=150.899 p=0.000<br>F(1,6)=8.868 p=0.025	
	COX-2/β-Actin	F(1,6)=23.753 p=0.003<br>F(1,6)=107.321 p=0.000	
(x)	LPS (ng/mg)	F(1,14)=38.001 p=0.000<br>F(1,14)=57.741 p=0.000	
(y)	Phylum level		
	Bacteroidetes	F(1,14)=5.974 p=0.028<br>F(1,14)=10.500 p=0.006	
	β-Proteobacteria	F(1,14)=4.634 p=0.049<br>F(1,14)=2.751 p=0.119	
	δ,γ-Proteobacteria	F(1,14)=7.384 p=0.017<br>F(1,14)=7.858 p=0.014	
	ε-Proteobacteria	F(1,14)=3.436 p=0.085	

		F(1,14)=5.020 p=0.042
	Firmicutes	F(1,14)=28.234 p=0.000<br>F(1,14)=17.930 p=0.001

Figure 7			
NC&EC, EC&EJ			
(b)	EPM	OT (%)	OE (%)
		F(1,14)=110.31 p=0.000<br>F(1,14)=29.816 p=0.000	F(1,14)=381.619 p=0.000<br>F(1,14)=71.906 p=0.000
(c)	MB (%)	F(1,14)=310.116 p=0.000<br>F(1,14)=12.947 p=0.003	
(d)	LDT	TL (%)	NT
		F(1,14)=898.78 p=0.000<br>F(1,14)=81.661 p=0.000	F(1,14)=81.038 p=0.000<br>F(1,14)=5.209 p=0.039
(e)	p-p65/p65	F(1,6)=16.497 p=0.007<br>F(1,6)=30.190 p=0.002	
	BDNF/β-Actin	F(1,6)=65.025 p=0.000<br>F(1,6)=47.190 p=0.000	
	Claudin-5/β-Actin	F(1,6)=9.994 p=0.020<br>F(1,6)=9.765 p=0.020	
(f)	TNF-α (pg/mg)	F(1,14)=11.542 p=0.004<br>F(1,14)=13.524 p=0.002	
(g)	IL-1β (pg/mg)	F(1,14)=154.991 p=0.000<br>F(1,14)=54.353 p=0.000	
(h)	IL-6 (pg/mg)	F(1,14)=27.217 p=0.000<br>F(1,14)=31.537 p=0.000	
(j)	Corticosterone (ng/mL)	F(1,14)=264.831 p=0.000<br>F(1,14)=95.253 p=0.000	
(k)	TNF-α (pg/mL)	F(1,14)=309.065 p=0.000<br>F(1,14)=645.721 p=0.000	
(l)	IL-1β (pg/mL)	F(1,14)=966.156 p=0.000<br>F(1,14)=689.787 p=0.000	
(m)	IL-6 (pg/mL)	F(1,14)=3848.148 p=0.000<br>F(1,14)=456.582 p=0.000	
(n)	LPS (ng/mL)	F(1,14)=46.084 p=0.000<br>F(1,14)=49.137 p=0.000	
(o)	Weight gain (g)	F(1,14)=126.962 p=0.000<br>F(1,14)=60.480 p=0.000	
(p)	Macroscopic score	F(1,14)=92.237 p=0.000<br>F(1,14)=15.000 p=0.002	
(q)	Colon length (cm)	F(1,14)=73.263 p=0.000<br>F(1,14)=27.961 p=0.000	
(r)	MPO activity (μU/mg)	F(1,14)=99.536 p=0.000<br>F(1,14)=70.680 p=0.000	
(s)	IL-6 (pg/mg)	F(1,14)=123.606 p=0.000<br>F(1,14)=39.526 p=0.000	
(t)	TNF-α (pg/mg)	F(1,14)=200.316 p=0.000<br>F(1,14)=149.318 p=0.000	
(u)	IL-1β (pg/mg)	F(1,14)=1145.022 p=0.000<br>F(1,14)=187.951 p=0.000	
(v)	IL-10 (pg/mg)	F(1,14)=51.830 p=0.000<br>F(1,14)=173.178 p=0.000	
(w)	p-p65/p65	F(1,6)=20.313 p=0.004<br>F(1,6)=15.912 p=0.007	
	iNOS/β-Actin	F(1,6)=36.301 p=0.001	

		F(1,6)=18.405 p=0.005
	COX-2/β-Actin	F(1,6)=107.431 p=0.000<br>F(1,6)=24.861 p=0.002
(x)	LPS (ng/mg)	F(1,14)=100.526 p=0.000<br>F(1,14)=286.352 p=0.000
(y)	Phylum level	
	Bacteroidetes	F(1,14)=4.268 p=0.058<br>F(1,14)=3.230 p=0.094
	β-Proteobacteria	F(1,14)=12.615 p=0.003<br>F(1,14)=11.943 p=0.004
	δ,γ-Proteobacteria	F(1,14)=8.932 p=0.010<br>F(1,14)=7.859 p=0.014
	ε-Proteobacteria	F(1,14)=0.575 p=0.461<br>F(1,14)=2.775 p=0.118
	Firmicutes	F(1,14)=3.192 p=0.096<br>F(1,14)=0.764 p=0.397

Figure S1		
NC&IS		
(b)	IL-1β (pg/mL)	F(1,14)=355.129 p=0.000
(c)	Weight gain (g)	F(1,14)=122.327 p=0.000
(d)	Macroscopic score	F(1,14)=65.258 p=0.000
(e)	IL-6 (pg/mg)	F(1,14)=414.934 p=0.000
(f)	TNF-α (pg/mg)	F(1,14)=1114.055 p=0.000
(g)	IL-1β (pg/mg)	F(1,14)=1168.241 p=0.000
(h)	IL-10 (pg/mg)	F(1,14)=202.862 p=0.000
(i)	Phylum level	
	Bacteroidetes	F(1,14)=4.801 p=0.046
	β-Proteobacteria	F(1,14)=6.023 p=0.028
	δ,γ-Proteobacteria	F(1,14)=7.384 p=0.017
	ε-Proteobacteria	F(1,14)=3.436 p=0.085
	Firmicutes	F(1,14)=28.207 p=0.000

Figure S2		
FNC&FIS		
(b)	IL-1β (pg/mL)	F(1,14)=425.171 p=0.000
(c)	Weight gain (g)	F(1,14)=12.334 p=0.003
(d)	Macroscopic score	F(1,14)=65.258 p=0.000
(e)	IL-6 (pg/mg)	F(1,14)=10.814 p=0.005
(f)	TNF-α (pg/mg)	F(1,14)=57.048 p=0.000
(g)	IL-1β (pg/mg)	F(1,14)=1081.204 p=0.000
(h)	IL-10 (pg/mg)	F(1,14)=43.914 p=0.000
(i)	Phylum level	
	Bacteroidetes	F(1,14)=19.179 p=0.001
	β-Proteobacteria	F(1,14)=10.780 p=0.005
	δ,γ-Proteobacteria	F(1,14)=17.385 p=0.001
	ε-Proteobacteria	F(1,14)=5.020 p=0.042
	Firmicutes	F(1,14)=19.864 p=0.001

Figure S3		
NC&EC		
(b)	IL-1β (pg/mL)	F(1,14)=966.156 p=0.000
(c)	Weight gain (g)	F(1,14)=126.962 p=0.000
(d)	Macroscopic score	F(1,14)=92.237 p=0.000

(e)	IL-6 (pg/mg)	F(1,14)=123.606 p=0.000
(f)	TNF-α (pg/mg)	F(1,14)=200.316 p=0.000
(g)	IL-1β (pg/mg)	F(1,14)=1145.022 p=0.000
(h)	IL-10 (pg/mg)	F(1,14)=51.830 p=0.000
(i)	Phylum level	
	Bacteroidetes	F(1,14)=4.235 p=0.059
	β-Proteobacteria	F(1,14)=12.615 p=0.003
	δ,γ-Proteobacteria	F(1,14)=8.932 p=0.010
	ε-Proteobacteria	F(1,14)=0.575 p=0.461
	Firmicutes	F(1,14)=3.190 p=0.096

Figure S4		
NC&EL5, EL10, EL50		
EPM	OT (%)	OE (%)
0 day	F(1,14)=0.031 p=0.863<br>F(1,14)=3.220 p=0.094<br>F(1,14)=0.328 p=0.576	F(1,14)=3.777 p=0.072<br>F(1,14)=1.957 p=0.184<br>F(1,14)=2.831 p=0.115
10th day	F(1,14)=48.638 p=0.000<br>F(1,14)=822.77 p=0.000<br>F(1,14)=1233.03 p=0.000	F(1,14)=24.890 p=0.000<br>F(1,14)=63.601 p=0.000<br>F(1,14)=2637.673 p=0.000

Figure S5		
NC&IS, IS&IJ, IS&IP		
	OT (%)	OE (%)
EPM	F(1,14)=203.35 p=0.000<br>F(1,14)=300.71 p=0.000<br>F(1,14)=275.754 p=0.000	F(1,14)=754.038 p=0.000<br>F(1,14)=411.568 p=0.000<br>F(1,14)=110.454 p=0.000

Figure S6		
EC, EL		
(b)	TNF-α (pg/mL)	F(1,8)=2.549 p=0.149<br>F(1,8)=7.561 p=0.025
(c)	IL-6 (pg/mL)	F(1,8)=21.904 p=0.002<br>F(1,8)=15.773 p=0.004
(e)	TNF-α (pg/mL)	F(1,8)=121.049 p=0.000<br>F(1,8)=301.706 p=0.000
(f)	IL-6 (pg/mL)	F(1,8)=477.664 p=0.000<br>F(1,8)=375.404 p=0.000

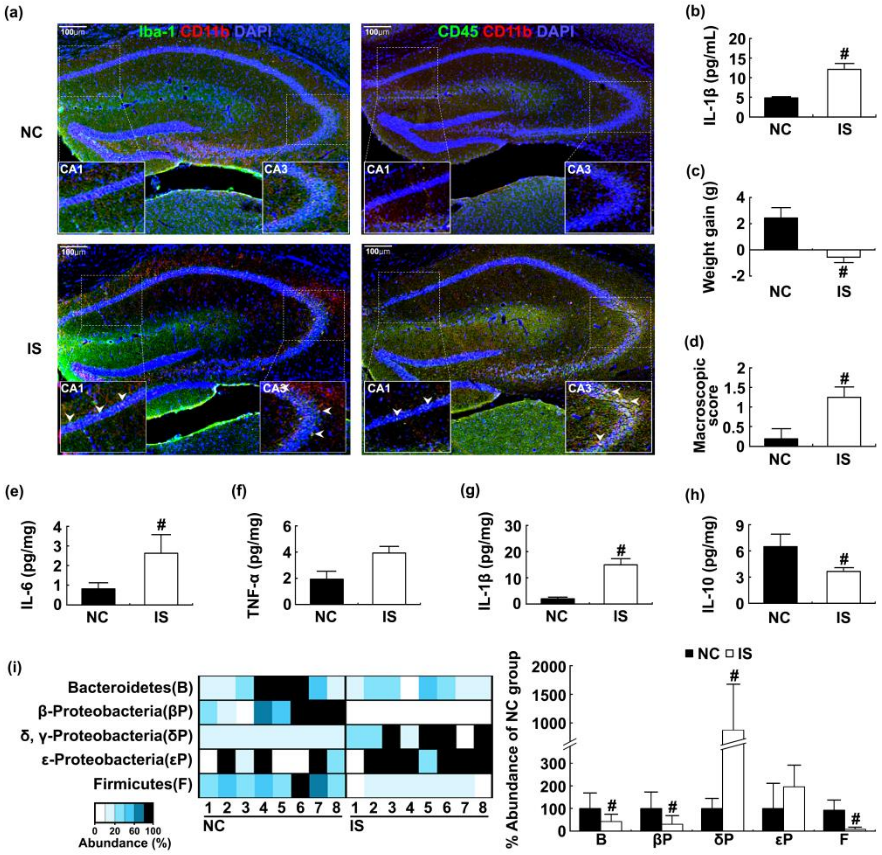

Supplement Figure S1. Immobilization stress induced anxiety-like behaviors and colitis in mice. Exposure of mice to immobilization stress (IS) performed once a day for 10 days. Control mice (NC) were not treated with IS. Microglia (Iba-1$^+$/CD11b$^+$) and monocytes (CD11b$^+$/CD11b$^+$) were assayed in the hippocampus by a confocal microscope (a). Blood IL-1β levels (b) were analyzed by using ELISA kit. Colitis markers body weight gain (c), macroscopic score (d), IL-6 (e), TNF-α (f), IL-1β (g), and IL-10 (h) levels were measured in the colon. Gut microbiota composition were measured by qPCR (i). The bacterial abundance was indicated as % of NC. All data were expressed as mean ± SD (n = 8). $^{\#}$ $p < 0.05$ vs. normal control (NC) group.

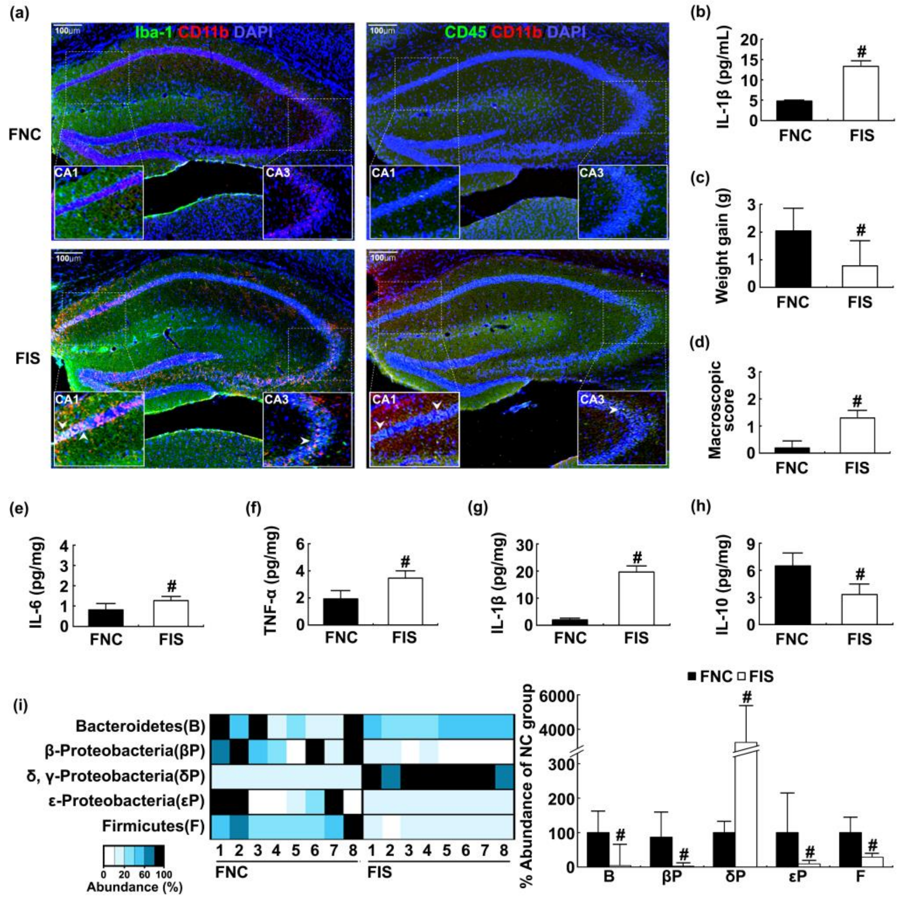

Supplement Figure S2. Fecal microbiota of immobilization stress-treated mice (FIS) induced anxiety-like behaviors and colitis in mice. FIS and FNC (fecal microbiota of IS-untreated control mice) were orally administered once a day for 5 days. Microglia (Iba-1^{+}/$CD11b^{+}$) and monocytes ($CD11b^{+}$/$CD11b^{+}$) were assayed in the hippocampus by a confocal microscope (a). Blood IL-1β levels (b) were analyzed by using ELISA kit. Colitis markers body weight gain (c), macroscopic score (d), IL-6 (e), TNF-α (f), IL-1β (g), and IL-10 (h) levels were measured in the colon. Gut microbiota composition were measured by qPCR (i). FNC and FIS in figures indicate groups treated with fecal microbiota of control and IS-treated mice (suspended in 1% dextrose), respectively. The bacterial abundance was indicated as % of FNC. All data were expressed as mean ± SD (n = 8). $^{\#}$ $p < 0.05$ vs. FNC

group.

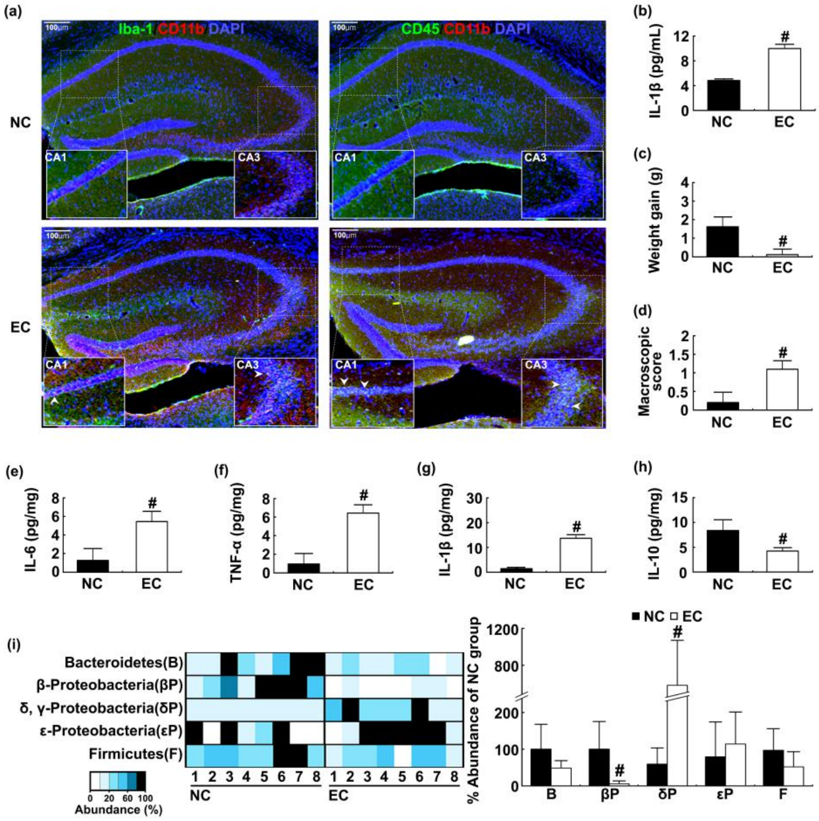

Supplement Figure S3. *Escherichia coli* (EC) induced anxiety-like behaviors and colitis in mice. Exposure of mice to immobilization stress (IS) performed once a day for 10 days. Control mice (NC) were treated with 1% dextrose alone instead of EC. Blood TNF-α (a) and IL-1β levels (b) were analyzed by using ELISA kit. Microglia (Iba-1$^+$/CD11b$^+$) and monocytes (CD11b$^+$/CD11b$^+$) were assayed in the hippocampus by a confocal microscope (a). Blood IL-1β levels (b) were analyzed by using ELISA kit. Colitis markers body weight gain (c), macroscopic score (d), IL-6 (e), TNF-α (f), IL-1β (g), and IL-10 (h) levels were measured in the colon. Gut microbiota composition were measured by qPCR (i). NC and EC in figures indicate groups treated with vehicle alone (1% dextrose) and *E. coli*, respectively. The bacterial abundance was indicated as % of NC. All data were expressed as mean ± SD (n = 8). $^{\#}$ $p < 0.05$ vs. NC group.

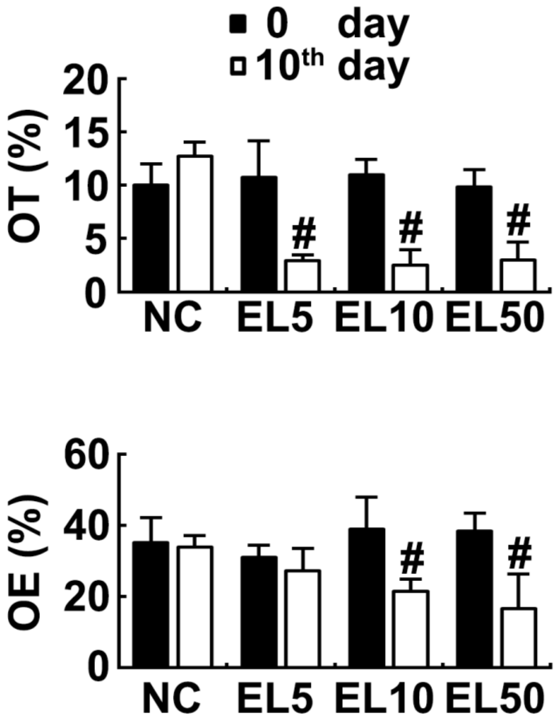

Supplement Figure S4. LPS purified from *Escherichia coli* (EL) caused anxiety in mice. Anxiety-like behaviors were measured on the 10th day after treatment with LPS in the EPM. LPS (L5, 5 μg/kg; L10, 10 μg/kg; and L50, 50 μg/kg) was intraperitoneally injected once a day for 5 days. Control group (NC) was not treated with EL. EPM task (time spent in open arms [OT] and open arm entries [OE]) was measured on the 10th day after the initial LPS treatment. All data were expressed as mean ± SD (n = 8). $^{\#}$ $p < 0.05$ vs. NC group.

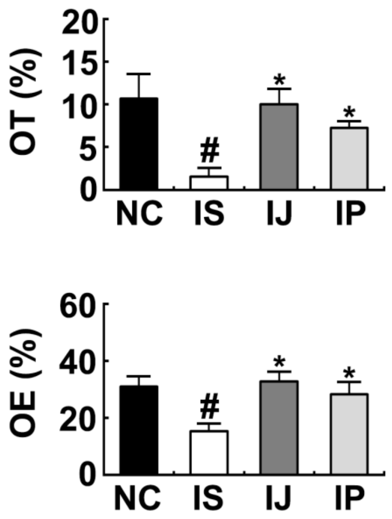

Supplement Figure S5. Oral administration of *Lactobacilli* alleviated immobilization stress (IS)-induced anxiety in mice. Anxiety-like behaviors were measured on the 10th day after the initial treatment with IS in the EPM. IS was treated for 10 days. *Lactobacillus johnsonii* and *Lactobacillus plantarum* (1×10^9 CFU/mouse) were isolated from the feces of normal control mice and orally administered once a day for 5 days from the 6th day after the initial IS treatment. EPM task (time spent in open arms [OT] and open arm entries [OE]) was measured on the 10th day after the initial LPS treatment. NC, IS, IJ, and IP in figures indicate groups treated with vehicle alone (1% dextrose) in control mice, vehicle in IS-treated mice, *L. johnsonii* in IS-treated mice, and *L. plantarum* in IS-treated mice, respectively. All data were expressed as mean ± SD (n = 8). $^{\#}p < 0.05$ vs. NC group. $^{*}p < 0.05$ vs. IS-treated group (IS).

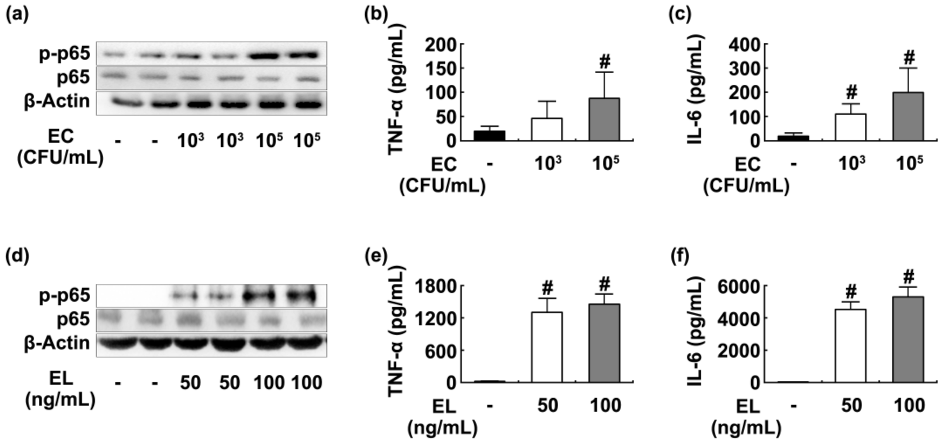

(g)

EC results after 24 hours

Tests	Substrate	Reaction	(-) Results	(+) Results	EC
ONPG	ONPG	β-galactosidase	colorless	yellow	+
ADH	L-arginine	Arginine dihydrolase	yellow	red/orange	-
LDC	L-lysine	Lysine decarboxylase	yellow	red/orange	+
ODC	L-ornithine	Ornithine decarboxylase	yellow	red/orange	+
CIT	trisodium citrate	Citrate Utilization	pale to green/yellow	blue-green/blue	+
H_2S	sodium thiosulfate	H_2S production	colorless/gray	black deposit	-
URE	Urea	Urea hydrolysis	yellow	red/orange	-
TDA	L-tryptophane	deaminase	yellow	brown-red	-
IND	L-tryptophane	Indole production	yellow	red (in 2 min)	+
VP	sodium pyruvate	acetoin production	colorless	pink/red (in 10 min)	-
GEL	Charcoal gelatin	gelatinase	no diffusion of black	black diffuse	-
GLU	D-glucose	fermentation/oxidation	blue/blue-green	yellow	+
MAN	D-mannitol	fermentation/oxidation	blue/blue-green	yellow	+
INO	Inositol	fermentation/oxidation	blue/blue-green	yellow	-
SOR	D-sorbitol	fermentation/oxidation	blue/blue-green	yellow	+
RHA	L-rhamnose	fermentation/oxidation	blue/blue-green	yellow	+
SAC	D-sucrose	fermentation/oxidation	blue/blue-green	yellow	-
MEL	D-melibiose	fermentation/oxidation	blue/blue-green	yellow	+
AMY	Amygdalin	fermentation/oxidation	blue/blue-green	yellow	-
ARA	L-arabinose	fermentation/oxidation	blue/blue-green	yellow	+
OX	oxidase	oxidase	colorless/yellow	violet	-
NO_2	potassium nitrate	NO_2 production	yellow	red	+
N_2		reduction to N_2 gas	orange-red	yellow	-
MOB	API M Medium or microscope	motility	non-motile	motile	+
McC	MacConkey medium	growth	absence	presence	+
OF-O	glucose (API OF Medium)	oxidation : exposed to the air	green	yellow	+
OF-F	glucose (API OF Medium)	fermentation : under mineral oil	green	yellow	+

Supplement Figure S6. *E. coli* (EC) and its LPS (EL) activated macrophages. Effect of EC on NF-κB activation (a) and TNF-α (b) and IL-6 expression (c). Effect of EL on NF-κB activation (d) and TNF-α (e) and IL-6 expression (f). The biochemical profile of EC, assessed by API 20E kit (g). Macrophage cells (0.5×10^6) were incubated with vehicle, EC, or LPS for 90 min (NF-κB and β-actin) or 20 h (for cytokines). Proteins were measured by immunoblotting. All values are expressed as mean ± SD (n = 4). $^{\#}p < 0.05$ vs. vehicle-treated group.

The 16S rDNA sequence of *E. coli* (below) isolated in the present study exhibited 99% homology with that of *E. coli* SABA3 (pubmed).

Bacteria	Sequence
E. coli isolated in the present study	CTTGCTCTTGGGTGACGAGTGGCGGACGGGTGAGTAATGTCTGGGAAACTGCCCGA<br>TGGAGGGGGATAACTACTGGAAACGGTAGCTAATACCGCATAACGTCGCAAGACCA<br>AAGAGGGGGACCTTCGGGCCTCTTGCCATCGGATGTGCCCAGATGGGATTAGCTTGT<br>AGGTGAGGTAACGGCTCACCTAGGCGACGATCCCTAGCTGGTCTGAGAGGATGACC<br>AGCCACACTGGAACTGAGACACGGTCCAGACTCCTACGGGAGGCAGCAGTGGGGA<br>ATATTGCACAATGGGCGCAAGCCTGATGCAGCCATGCCGCGTGTATGAAGAAGGCCT<br>TCGGGTTGTAAAGTACTTTCAGCGGGGAGGAAGGGAGTAAAGTTAATACCTTTATTC<br>ATTGACGTTACCCGCAGAAGAAGCACCGGCTAACTCCGTGCCAGCAGCCGCGGTAA<br>TACGGAGGGTGCAAGCGTTAATCGGAATTACTGGGCGTAAAGCGCACGCAGGCGGT<br>TTGTTAAGTCAGATGTGAAATCCCCGGGCTCAACCTGGGAACTGCATCTGATACTGG<br>CAAGCTTGAGTCTCGTAGAGGGGGGTAGAATTCCAGGTGTAGCGGTGAAATGCGTA<br>GAGATCTGGAGGAATACCGGTGGCGAAGGCGGCCCCCTGGACGAAGACTGACGCTC<br>AGGTGCGAAAGCGTGGGGAGCAAACAGGATTAGATACCCTGGTAGTCCACGCCGTA<br>AACGATGTCGACTTGGAGGTTGTGCCCTTGAGGCGTGGCTTCCGGAGCTAACGCGTT<br>AAGTCGACCGCCTGGGGAGTACGGCCGCAAGGTTAAAACTCAAATGAATTGACGGG<br>GGCCCGCACAAGCGGTGGAGCATGTGGTTTAATTCGATGCAACGCGAAGAACCTTA<br>CCTGGTCTTGACATCCACGGAAGTTTTCAGAGATGAGAATGTGCCTTCGGGAACCGT<br>GAGACAGGTGCTGCATGGCTGTCGTCAGCTCGTGTTGTGAAATGTTGGGTTAAGTCC<br>CGCAACGAGCGCAACCCTTATCCTTTGTTGCCAGCGGTCCGGCCGGGAACTCAAAG<br>GAGACTGCCAGTGATAAACTGGAGGAAGGTGGGGATGACGTCAAGTCATCATGGCC<br>CTTACGACCAGGGCTACACACGTGCTACAATGGCGCATACAAAGAGAAGCGACCTC<br>GCGAGAGCAAGCGGACCTCATAAAGTGCGTCGTAGTCCGGATTGGAGTCTGCAACT<br>CGACTCCATGAAGTCGGAATCGCTAGTAATCGTGGATCAGAATGCCACGGTGAATAC<br>GTTCCCGGGCCTTGTACACACCGCCCGTCACACCATGGGAGTGGGTTGCAAAAGA<br>AGTAGGTAGCTTAACCTTCGGGAGGGCGCTTACCACTTTGTGATTCATGACTGGGGT<br>GAATCT

Accession	Description	Max score	Total score	Query cover	E value	Max ident
KY305421.1	*Escherichia coli* strain SABA3 16S ribosomal RNA gene, partial sequence	2562	2562	99%	0	99%
CP010134.1	*Escherichia coli* strain D1, complete genome	2562	17876	99%	0	99%
